# Serotonin depletion amplifies migraine susceptibility through trigeminal ganglion neurons sensitization: Insights from patch clamp recordings in rat model

**DOI:** 10.1371/journal.pone.0354679

**Published:** 2026-08-06

**Authors:** Sirikorn Vongseenin, Sekh Thanprasertsuk, Naeemah Ha-ji-a-sa, Saknan Bongsebandhu-phubhakdi

**Affiliations:** 1 Department of Physiology, Faculty of Medicine, Chulalongkorn University, Bangkok, Thailand; 2 Cognitive Clinical and Computational Neuroscience Center of Excellence, Chulalongkorn University, Bangkok, Thailand; 3 Chula Neuroscience Center, King Chulalongkorn Memorial Hospital, Thai Red Cross Society, Bangkok, Thailand; Instituto Butantan, BRAZIL

## Abstract

Serotonin (5-HT) depletion is a critical driver of migraine pathophysiology, yet its precise impact on trigeminal ganglion (TG) neurons remains unclear. This study investigates how 5-HT depletion shapes the “**intrinsic neuronal susceptibility”** of the first-order TG neurons by analyzing the electrophysiological properties of small-to-medium (SM) and large (L)-sized TG neurons in a rat model using patch-clamp recordings. Our findings reveal that 5-HT depletion significantly heightens the intrinsic susceptibility of TG neurons, with the electrophysiological disparities between SM and L-sized neurons acting as aggravating factors that intensify peripheral sensitization. Although this study focuses on intrinsic neuronal properties, our findings raise the possibility that 5-HT depletion may indirectly influence the functional balance between pro- and anti-nociceptive 5-HT receptor subtypes within the trigeminovascular system, a hypothesis that warrants further investigation. Functionally, this increased neuronal excitability may predispose TG neurons to heightened responsiveness, potentially contributing to hyperalgesia and altered somatosensory processing. By elucidating the role of serotonin in maintaining sensory homeostasis, this study provides critical insights into migraine pathogenesis and highlights the potential for targeted serotonergic therapies to mitigate excessive trigeminovascular activation.

## Introduction

Serotonin (5-HT) is a key neurotransmitter that plays a vital role in regulating both central and peripheral nervous system functions. Its depletion has been strongly associated with the pathogenesis of several debilitating neurological disorders, including Parkinson’s disease [1], anxiety, major depressive disorder [2], sleep disturbances [3], and migraine [4]. In migraine patients, 5-HT levels are significantly reduced during attacks, and their restoration has been shown to mitigate pain [4,5]. Substantial evidence highlights the pivotal role of 5-HT in migraine pathophysiology, influencing both electrophysiological and molecular mechanisms through the peripheral [6] and central sensitization [7–9] of the trigeminovascular pathway. However, the precise mechanisms by which 5-HT modulates this pathway remain incompletely understood.

Migraine is a multifactorial disorder characterized by diverse clinical manifestations and variable susceptibility among individuals [10]. While genetic, environmental, and lifestyle factors [11–14] have been implicated, the intrinsic properties of primary sensory neurons that may underlie this variability remain poorly defined. In this context, we introduce the concept of **“intrinsic neuronal susceptibility”** of the trigeminovascular system, referring to the inherent excitability and responsiveness of TG neurons at the cellular level. This susceptibility reflects the baseline propensity of TG neurons to undergo sensitization, whereby 5-HT depletion may enhance overall neuronal susceptibility and predispose neurons to exaggerated responses to nociceptive stimuli. Therefore, we aimed to define intrinsic neuronal susceptibility at the cellular level by characterizing the electrophysiological properties of first-order TG neurons under 5-HT depleted conditions.

The trigeminovascular pathway, a central component of migraine pathophysiology, involves multiple levels of processing, with TG neurons serving as first-order neurons. These neurons can be broadly classified into small-to-medium (SM) and large (L) subtypes, which differ in their functional roles in nociceptive and somatosensory signaling. While SM neurons are predominantly nociceptive [15,16], L neurons exhibit more heterogeneous functions, including both nociceptive and non-nociceptive properties [17]. Electrophysiological features, particularly action potential (AP) waveform characteristics, are widely used to distinguish these functional phenotypes [17–20]. Recent identification of large-sized Ah-type myelinated nociceptive neurons further highlights the complexity and dynamic nature of TG neuron involvement in pain processing [21,22].

Peripheral sensitization of the first-order TG neurons is a key mechanism underlying migraine pain and is closely linked to serotonergic modulation. Reduced 5-HT levels may diminish inhibitory control over nociceptive signaling and facilitate the activity of pro-nociceptive mediators such as CGRP and nitric oxide [23–25]. In addition, altered activation of 5-HT receptor subtypes may further influence the balance between excitatory and inhibitory signaling within the trigeminovascular system [4,26,27]. Accumulating evidence suggests that 5-HT depletion, together with other neurotransmitters and neuropeptides, synergistically interacts with cortical spreading depression (CSD) to exacerbate sensitization, thereby promoting the transition toward central sensitization and increasing migraine susceptibility [6,28,29]. Clinically, peripheral sensitization manifests as hyperalgesia [30,31] and contributes to abnormal somatosensory processing, such as cutaneous allodynia [32], and may progress to central sensitization, leading to chronic migraine [33,34].

Our study seeks to explore the effects of 5-HT depletion on the altered baseline excitability of first-order TG neurons, along with the electrophysiological differences between SM and L-sized neurons. Using the patch-clamp recording technique, we analyze several parameters, including total spikes count, excitability parameters (rheobase, resting membrane potential, and threshold potential), and action potential (AP) properties (AP height, AP overshoot, AP rising, AP falling and AP duration). These parameters, along with their intergroup differences, are evaluated to understand how serotonin depletion influences the trigeminovascular pathway. Since total spikes generation in TG neurons is a key indicator of peripheral sensitization [9,35], investigating the correlation between total spikes and excitability parameters may offer valuable insights into the susceptibility to pain transmission. Elucidating these cellular-level alterations may provide mechanistic insight into how serotonergic dysfunction primes trigeminal ganglion neurons toward a hyperexcitable state, potentially facilitating trigeminovascular sensitization and increasing migraine susceptibility.

In this study, we aim to define this intrinsic neuronal susceptibility at the cellular level by characterizing the electrophysiological properties of first-order trigeminal ganglion neurons under serotonin-depleted conditions.

## Materials and methods

### Experimental animals and grouping

The animal experiments in this study were conducted in full compliance with the ethical guidelines set by the Animal Care and Use Committee of the Faculty of Medicine, Chulalongkorn University, Thailand (Approval No. 019/2561). The study followed the *Guide for the Care and Use of Laboratory Animals* (8th Edition, National Academies Press) and adhered to the ARRIVE reporting guidelines. Adult male Wistar rats weighing between 200 and 300 grams were procured from Nomura Siam International, Bangkok, Thailand, for use in all experiments. The rats were housed in stainless steel cages within a well-ventilated environment, maintained under a 12-hour light-dark cycle, with unrestricted access to food and water.

Rats were categorized into two primary groups: 5-HT-depleted rats and control rats. To induce 5-HT depletion, para-chlorophenylalanine (PCPA; Sigma Aldrich, CA, USA) was administered intraperitoneally at a dose of 100 mg/kg body weight, dissolved in physiological saline (0.9%), for three consecutive days. This protocol was adapted from previously validated studies and is widely recognized as a standardized method for inducing systemic 5-HT depletion [9]. PCPA, as an irreversible inhibitor of tryptophan hydroxylase, reduces serotonin synthesis globally across central and peripheral compartments [36]. Prior studies have demonstrated that repeated intraperitoneal PCPA treatment produces substantial reductions in circulating 5-HT levels, with accompanying central serotonergic depletion confirmed in related paradigms [37,38]. To categorize TG neuronal size, we defined small-to-medium-sized neurons as those with a diameter <30 µm and capacitance <50 pF, and large-sized neurons as those with a diameter ≥30 µm and capacitance ≥50 pF, ensuring classification accuracy. Based on 5-HT depletion status and neuronal size, TG neurons were divided into four groups: small-to-medium (PCPA-SM) and large-sized (PCPA-L) neurons from 5-HT-depleted rats, and small-to-medium (control-SM) and large-sized (control-L) neurons from control rats.

### TG removal and primary culture

The processes of TG removal and culture were carried out following previously established protocols [35,39]. Rats from both experimental groups were sacrificed 24 hours after receiving PCPA on the third day, using intraperitoneal thiopental injection (70 mg/kg body weight), followed by decapitation. Trigeminal ganglia were collected 24 hours after the final PCPA injection, and electrophysiological recordings were performed 18–24 hours after primary culture. The TG from both sides were carefully extracted and transferred into 35-mm culture dishes containing ice-cold Hank’s balanced salt solution (HBSS) supplemented with penicillin/streptomycin (10–20 µL; 10,000 U penicillin and 10 mg streptomycin/mL).

The ganglia were washed twice with HBSS, minced into small fragments using a sterile razor blade in 1 mL of HBSS, and transferred to a plastic tube. Collagenase (100 µL; 2 mg/mL) and dispase (200 µL; 50 U/mL) were added to the sample, which was then filtered with a 0.22-µm filter and incubated in a 37°C water bath for 20 minutes. After incubation, the sample was centrifuged at 400 g RCF for 1 minute to remove the supernatant, followed by the addition of papain enzyme. The mixture was incubated again in a 37°C water bath for 20 minutes. Subsequently, 2 mL of L-15 medium was added to the sample, which was centrifuged for 8 minutes. The cells were then washed with 400 µL of F-12 complete medium, plated onto laminin/PDL-coated dishes, and placed in an incubator (37°C, 5% CO_2_) for 3 hours.

After this initial incubation period, the F-12 complete medium was replaced twice, and the samples were maintained in the incubator for an additional 18–24 hours before being subjected to the electrophysiological patch-clamp study. Neurons were confirmed to be attached by focusing on a single plane at the bottom of the dish, and any neurons appearing swollen or bloated were excluded from the study.

### Patch-clamp recording

Whole-cell patch-clamp recordings were conducted following the methodology described in prior studies [35,39]. Trigeminal ganglion (TG) neuron samples were placed in plastic chambers positioned on a microscope stand (Olympus BX51WI, Olympus, Japan). The neurons were continuously perfused with an external solution at a controlled flow rate of 1 mL/min and maintained at 25°C. The external solution consisted of 145 mM NaCl, 5 mM KCl, 2 mM CaCl2, 1 mM MgCl2, 10 mM D-glucose, and 10% HEPES. Its pH was precisely adjusted to 7.40 using 1 M NaOH, and the osmolality was standardized to 320 ± 5 mOsm/kg with D-glucose.

Microelectrodes were fabricated with an outer diameter of 1.5 mm and an inner diameter of 0.86 mm (Sutter Instruments, Navato, CA, USA) and filled with an internal solution containing 140 mM K-gluconate, 1 mM CaCl_2_, 10 mM EGTA, 10 mM HEPES, and 10 mM ATP. The osmolality of the internal solution was calibrated to 290 ± 5 mOsm/kg using D-glucose. Patch pipettes were designed to have tip resistances ranging from 6 to 8 MΩ. The formation of a stable gigaseal, signifying successful neuron attachment, was confirmed by gently moving the electrode. During the recording process, electrophysiological activity of each neuron was continuously monitored.

Only neurons meeting strict inclusion criteria were selected for data analysis. These criteria included a resting membrane potential (RMP) more negative than −40 mV, a series resistance between 10 and 25 MΩ, and a capacitance below 200 pF, ensuring that the analyzed neurons exhibited normal electrophysiological properties [16]. Additionally, the diameter (µm) and morphology of each neuron were recorded.

The key electrophysiological parameters assessed included the total number of spikes which were counted based on the number of APs with peak membrane potentials exceeding 0 mV, excitability metrics such as rheobase (pA), threshold potential (mV), and resting membrane potential (mV), as well as action potential characteristics, including AP height (mV), AP overshoot (mV), AP rising time (ms), AP falling time (ms), and AP duration (ms). All recordings were meticulously conducted using an Axopatch 200B amplifier (Axon Instruments, CA, USA), with data acquisition performed using Clampex 10.2 software (Molecular Devices, CA, USA).

### Statistical analysis

Electrophysiological parameters were expressed as mean ± standard deviation. Initially, a one-way ANOVA was conducted to compare TG neuron sizes, while Pearson’s chi-square test was used to analyze bursting profiles. A linear correlation test was then performed to assess relationships between parameter pairs, with results presented as Pearson correlation coefficients and *p*-values. Subsequently, variations between groups for each parameter were evaluated. Data normality was first assessed using the Shapiro-Wilk test. If the *p*-value was < 0.05, indicating a non-normal distribution, the Kruskal-Wallis test was applied, followed by pairwise comparisons to identify intergroup differences. If the *p*-value was > 0.05, suggesting normal distribution, a one-way ANOVA was used, followed by Tukey’s post hoc test for multiple comparisons. Statistical significance was set at *p* < 0.05.

## Results

A total of 49 trigeminal ganglion (TG) neurons were collected and categorized into two primary groups based on whether the neurons were derived from PCPA-injected rats or non-injected controls: control neurons (n = 25) and PCPA neurons (n = 24). Each group was further subdivided according to neuronal size, using both diameter and capacitance criteria.This classification resulted in four experimental groups: control-SM(n = 13), control-L(n = 12), PCPA-SM(n = 11), and PCPA-L(n = 13). Key electrophysiological parameters, including the total number of spikes, excitability parameters (RMP, threshold potential, and rheobase), and action potential properties, were recorded and analyzed using statistical methods.

### Neuronal classification and baseline characteristics

We first examined the relationship between neuronal excitability parameters and spike generation to assess intrinsic susceptibility under 5-HT-depleted conditions. A one-way ANOVA test was conducted to compare the neuron sizes across the four groups, revealing significant differences in mean diameter consistent with the initial size-based grouping (Table 1, *p* < 0.001, one-way ANOVA test). However, the post-hoc Tukey’s test showed no significant changes in diameters between control-SM (24.33 ± 2.88 µm) and PCPA-SM (24.80 ± 3.00 µm) (Table 1, *p* = 1.000) or control-L (31.11 ± 6.47 µm) and PCPA-L (33.19 ± 4.24 µm) (Table 1, *p* = 0.639). Pearson’s chi-square test was then employed to compare the overall bursting patterns among all groups and between the control and PCPA-injected groups (control-SM vs. PCPA-SM and control-L vs. PCPA-L). The results, summarized in Table 1, revealed a significant difference in the proportion of single-spike and multiple-spike neurons across all groups (Table 1, *p* = 0.001, Pearson’s chi-square test), aligning with the basic property differences between these neuron types. However, no significant differences in bursting profiles were observed following 5-HT depletion induced by PCPA injection in both SM and L-sized neurons (Table 1, *p* = 0.772 and *p* = 0.490, respectively, Pearson’s chi-square test).

**Table 1:** Demographic data of the trigeminal ganglion neurons in the control-SM group, PCPA-SM group, control-L group and PCPA-L group.

Parameters	Control-SM	PCPA-SM	Control-L	PCPA-L	p-value^a^
Number	13	11	12	13	–
Size (μm)	24.33 ± 2.88	24.80 ± 3.00	31.11 ± 6.47	33.19 ± 4.24	<0.001
	*p*^a^=1.000		*p*^*a*^=0.639		
Single- spike/Multi -spike neurons	4/9	4/7	10/2	12/1	0.001
	*p*^b^=0.772		*p*^b^ =0.490		

^a^*p*-value ANOVA for diameter

^b^*p*-value derived from Pearson’s Chi-square test comparing the distribution of bursting profiles (single-spike vs. multiple-spike neurons) between groups.

### Correlation between excitability parameters and spike generation

**Fig 1:**
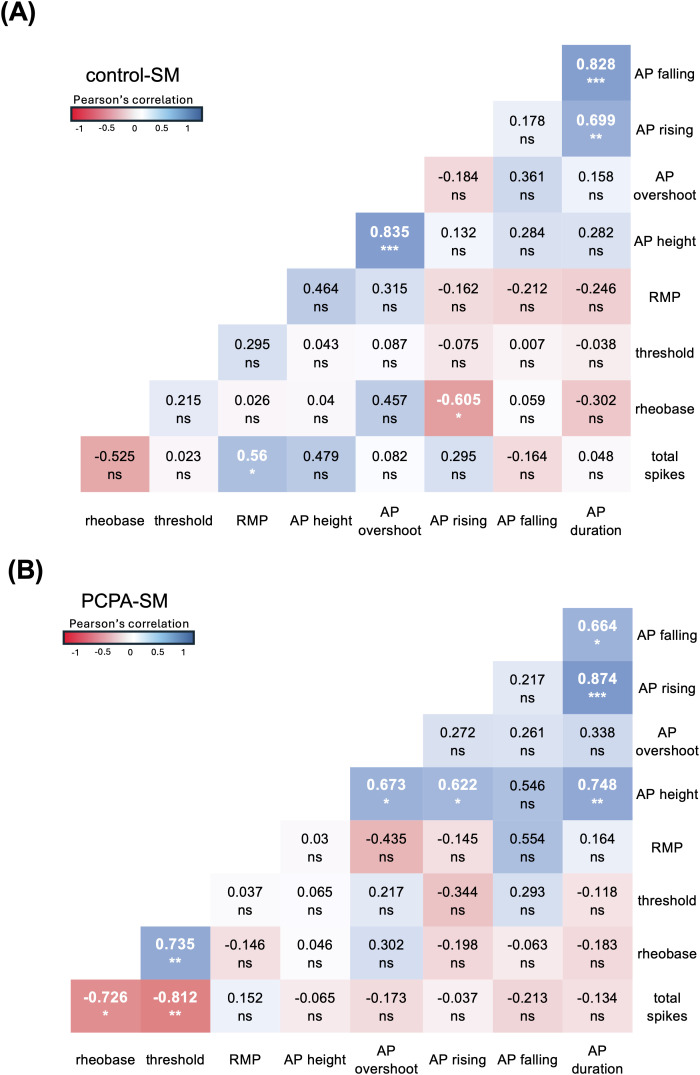
Heat map illustrating Pearson correlation coefficients and p-values from the linear correlation test between all parameters in (A) control-SM group (B) PCPA-SM group. The strength and direction of correlations are represented by a color gradient ranging from red (−1) to blue (1). Statistically significant correlations are marked with * (*p* < 0.05), ** (*p* < 0.01), and *** (*p* < 0.001).

**Fig 2:**
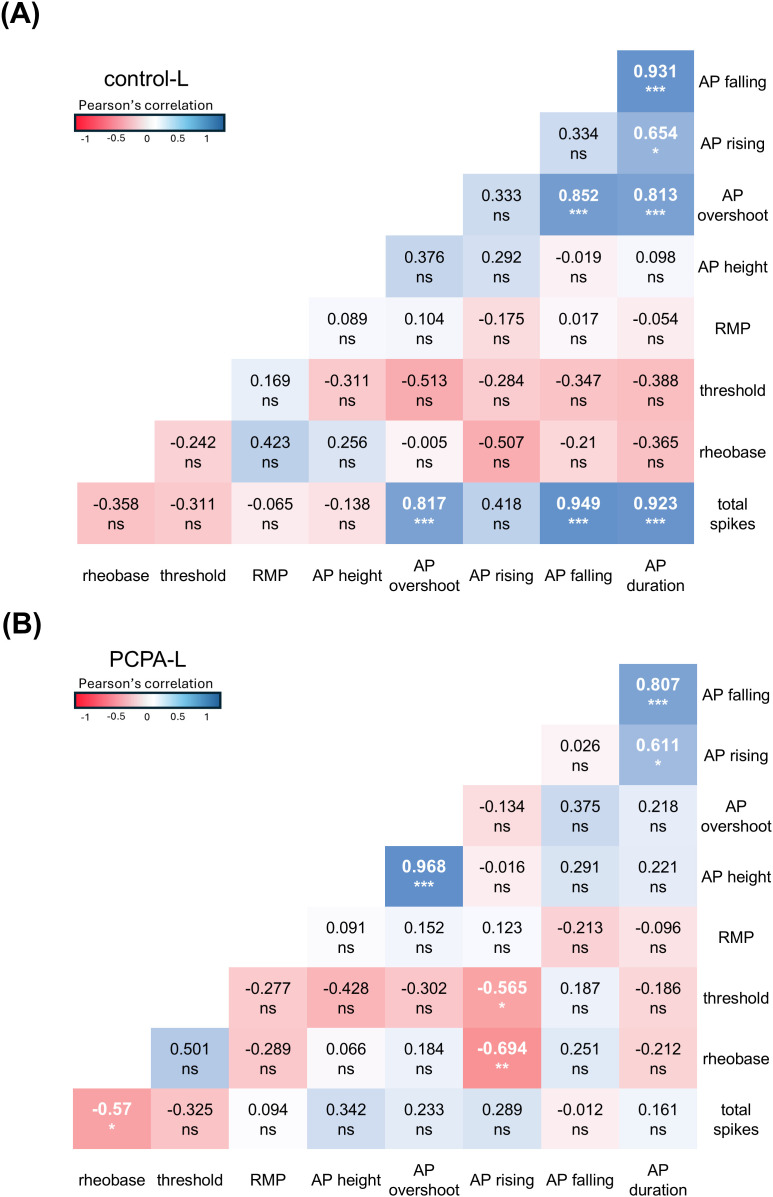
Heat map illustrating Pearson correlation coefficients and p-values from the linear correlation test between all parameters in (A) control-L group (B) PCPA-L group. The strength and direction of correlations are represented by a color gradient ranging from red (−1) to blue (1). Statistically significant correlations are marked with * (*p* < 0.05), ** (*p* < 0.01), and ***(*p* < 0.001).

We next assessed the relationship between neuronal firing and excitability parameters to evaluate changes in pain susceptibility across groups (Figs 1 and 2). In this study, we evaluated pain susceptibility by examining the correlation between the total number of spikes, an indicator of neuronal sensitization, and key excitability parameters, including RMP, threshold potential, and rheobase, across the four experimental groups (Figs 1 and 2). Notably, significant negative correlations were exclusively identified in the PCPA-treated neurons (Figs 1B and 2B). In the PCPA-SM group, total spikes showed a strong negative correlation with both rheobase (Fig 1B, Pearson correlation = −0.726, *p* = 0.011) and threshold potential (Fig 1B, Pearson correlation = −0.812, *p* = 0.002). Similarly, in the PCPA-L group, a moderate negative correlation was observed between total spikes and rheobase (Fig 2B, Pearson correlation = −0.57, *p* = 0.042). Conversely, no significant correlations were detected between total spikes and excitability parameters, including rheobase and threshold potential, in control neurons of either size (Figs 1A and 2A). Additionally, a moderate positive correlation was found between total spikes and RMP in the control-SM group (Fig 1A, Pearson correlation = 0.56, *p* = 0.047), a relationship that was absent in the PCPA-SM group (Fig 1B).

### Action potential properties and their relationship with neuronal firing

We next examined the relationships between AP parameters, reflecting inward current dynamics and nociceptive-related properties, and the excitability parameters across groups (Figs 1 and 2. In SM-sized neurons, no significant correlation was detected between total spikes and AP parameters (Figs 1A and 1B). In contrast, within the control-L group, strong positive correlations were observed between total spikes and AP parameters, including AP falling time (Fig 2A, Pearson correlation = 0.949, *p* < 0.001) and AP duration (Fig 2A, Pearson correlation = 0.923, *p* < 0.001). Additionally, a strong positive correlation was identified between total spikes and AP overshoot in the control-L group (Fig 2A, Pearson correlation = 0.817, *p* = 0.001). Notably, in the PCPA-L group, no significant or consistent correlation trends were detected between total spikes and any AP parameters (Fig 2B). There are also notable correlations between excitability parameters and AP parameters, particularly AP rising time. In the control-SM group, a moderate negative correlation was observed between rheobase and AP rising time (Fig 1A, Pearson correlation = −0.605, *p* = 0.028); however, this correlation disappeared following PCPA injection (Fig 1B). Conversely, in the PCPA-L group, new correlations between excitability parameters and AP parameters emerged when compared to the control-L group. Specifically, a moderate negative correlation was identified between threshold potential and AP rising time (Fig 2B, Pearson correlation = −0.565, *p* = 0.044), along with a strong negative correlation between rheobase and AP rising time (Fig 2B, Pearson correlation = −0.694, *p* = 0.008). Several significant correlations were also identified among AP parameters. A moderate-to-strong positive correlation was noted between AP duration and its components, AP rising time and AP falling time, across all groups (Figs 1 and 2). In the PCPA-SM group, compared to the control-SM group, a positive correlation was observed between AP height and AP duration parameters, including AP rising time (Pearson’s correlation = 0.622, *p* = 0.041) and AP duration (Fig 1, Pearson’s correlation = 0.748, *p* = 0.008). Additionally, in the control-L group, AP overshoot exhibited a strong positive correlation with AP falling time (Fig 2A, Pearson’s correlation = 0.852, *p* < 0.001) and AP duration (Fig 2A, Pearson’s correlation = 0.813, *p* = 0.001). Remarkably, these correlations disappeared in the PCPA-L group (Fig 2B).

### Intergroup differences in excitability and spike activity

**Fig 3:**
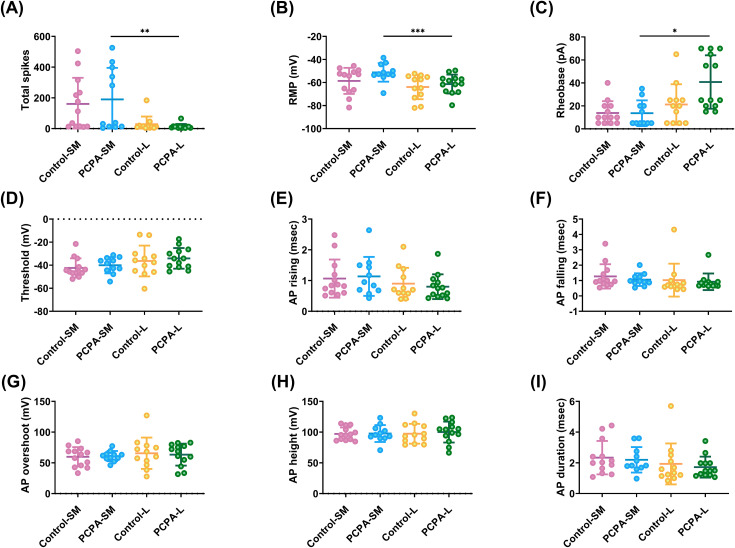
Excitability parameters and action potential parameters of trigeminal ganglion neurons in the control-SM group, the control-L group, the PCPA-SM group, and the PCPA-L group recorded by patch-clamp recording technique. **(A)** Total Spike **(B)** RMP **(C)** Rheobase **(D)** Threshold **(E)** AP rising **(F)** AP falling **(G)** AP overshoot **(H)** AP height **(I)** AP duration. The data shown represent mean ± SEM. Statistical analysis was performed using the Kruskal–Wallis test for panels (A–C, E–G, I) and one-way ANOVA for panels (D, H), followed by appropriate post-hoc multiple comparisons. Pairwise comparisons shown represent post-hoc analyses conducted after a significant omnibus test. Statistical significance is indicated as *p < 0.05, **p < 0.01, ***p < 0.001, and N.S. (not significant), based on the corresponding post-hoc pairwise comparisons.

Finally, we compared electrophysiological parameters across experimental groups to identify differences associated with 5-HT depletion and neuronal size. The Shapiro-Wilk test of normality guided the selection of the statistical test, with parameters exhibiting normal distribution (*p* > 0.05)—such as threshold potential and AP height—analyzed via one-way ANOVA, while non-normally distributed parameters, including total spikes, RMP, rheobase, AP overshoot, AP rising time, AP falling time, and AP duration, were analyzed using the Kruskal-Wallis test. The Kruskal-Wallis test revealed significant differences among the four groups in total spikes, RMP, and rheobase (Table 2, Figs 3A–3C, *p* = 0.001, 0.012 and 0.003, respectively). Pairwise comparisons identified remarkable distinctions between the PCPA-SM and PCPA-L groups. Specifically, the total spikes count was significantly higher in the PCPA-SM group (189.91 ± 205.63) compared to the PCPA-L group (13.69 ± 16.30) (Table 2, Fig 3A, *p* = 0.004, pairwise comparisons test). For excitability parameters, the PCPA-SM group displayed a more depolarized RMP (−51.25 ± 7.99 mV) compared to the PCPA-L group (−61.36 ± 8.21 mV) (Table 2, Fig 3B, *p* < 0.001, pairwise comparisons test). Additionally, the PCPA-SM group exhibited a notably lower rheobase (13.64 ± 11.20 pA) than the PCPA-L group (40.77 ± 23.26 pA) (Table 2, Fig 3C, *p* = 0.012, pairwise comparisons test). Furthermore, no significant differences in action potential properties were observed among any of the groups (Figs 3E–3I). And apparently, no significant differences in total spikes or excitability parameters were observed between the control-SM and control-L groups. There was also no significant difference in all parameters between control-group and PCPA-group in both SM and L sized neurons (Figs 3A–3I).

**Table 2:** Summary of the comparative electrophysiological parameters of the trigeminal ganglion neurons in the control-SM group, control-L group, PCPA-SM group and PCPA-L group (*p-value < 0.05).

Parameters	Control-SM (N = 13)	Control-L (N = 12)	PCPA-SM (N = 11)	PCPA-L (N = 13)	p-value Kruskal	p-value ANOVA
RMP (mV)	−58.60 ± 11.31	−63.89 ± 10.59	−51.25 ± 7.99	−61.36 ± 8.21	0.012^*^	–
Rheobase (pA)	13.85 ± 10.24	21.25 ± 17.60	13.64 ± 11.20	40.77 ± 23.26	0.003^*^	–
Threshold (mV)	−42.39 ± 8.38	−36.35 ± 13.39	−40.02 ± 7.05	−34.07 ± 8.94	–	0.152
Total Spikes	160.62 ± 169.93	28.58 ± 50.18	189.91 ± 205.63	13.69 ± 16.30	0.001^*^	–
AP height (mV)	97.12 ± 10.46	97.36 ± 16.11	97.75 ± 13.77	100.12 ± 17.05		0.950
AP overshoot (mV)	59.83 ± 15.78	65.62 ± 25.50	61.01 ± 8.30	63.36 ± 17.82	0.863	–
AP rising (msec)	1.06 ± 0.62	0.90 ± 0.51	1.13 ± 0.63	0.80 ± 0.40	0.364	–
AP falling (msec)	1.27 ± 0.79	1.03 ± 1.07	1.06 ± 0.41	0.92 ± 0.54	0.067	–
AP duration (msec)	2.34 ± 1.09	1.93 ± 1.33	2.20 ± 0.83	1.72 ± 0.68	0.168	–

## Discussion

Our experiments uncover critical findings that highlight the heightened intrinsic electrophysiological properties of SM- and L-sized TG neurons following 5-HT depletion. Notably, correlation analysis revealed a negative relationship between total spikes and RMP in both the PCPA-SM and PCPA-L groups, with a stronger correlation observed in the former. Furthermore, electrophysiological disparities between SM- and L-sized neurons, as demonstrated by the Kruskal-Wallis test, showed that PCPA-SM neurons exhibited a higher RMP, lower rheobase, and lower threshold compared to PCPA-L neurons. It is important to note that our study specifically focuses on intrinsic electrophysiological properties of trigeminal ganglion neurons, and does not directly address molecular or receptor-level mechanisms underlying serotonergic modulation.

In migraine, the specific role of 5-HT depletion in modulating the trigeminovascular system remains unclear. However, previous studies have extensively explored its effects and identified its potential link to heighten the neuronal susceptibility of trigeminovascular pathway, thereby inducing the sensitization process. At the cortical level, 5-HT depletion increases the frequency and width of CSD waves, thereby enhancing cortical neuron excitability [7–9]. In the trigeminal nucleus caudalis (TNC) and cortex, 5-HT depletion has been associated with an increase in Fos-immunoreactive neurons, suggesting elevated nociceptive signaling [9]. In trigeminal ganglion (TG) neurons, 5-HT depletion combined with CSD induces peripheral sensitization and subsequently promotes central sensitization by increasing the excitability of small-to-medium (SM) sized TG neurons [6].

Consistent with these findings, our study demonstrated heightened peripheral sensitization under 5-HT-depleted conditions, as evidenced by increased neuronal hyperexcitability in the first order TG neurons from the fundamental electrophysiogical level. However, the Pearson’s Chi-square test showed no significant changes in the overall bursting profile (single/multiple spike) following PCPA injection. Correlation analysis revealed a strong negative relationship between rheobase and total spikes in the PCPA-SM group, indicating significantly increased neuronal susceptibility in SM-sized TG neurons. A moderately negative correlation between rheobase and total spikes was also observed in the PCPA-L group, suggesting that 5-HT depletion enhances the intrinsic susceptibility in both SM and L-sized nociceptive TG neurons. Additionally, under the 5-HT depleted conditions, the positive correlation between AP height and AP duration parameters (AP rising time and AP duration) observed in the PCPA-SM group suggests an increase in inward current coupled with disrupted AP repolarization, thereby highlighting heightened neuronal excitability [9]. This combination further exacerbates the susceptibility of TG neurons. This heightened pain susceptibility likely facilitates peripheral sensitization, which, in turn, drives central sensitization through the release of pain and inflammatory mediators. Previous molecular studies provide further support for this mechanism. Under normal conditions, 5-HT co-released with calcitonin gene-related peptide (CGRP) and nitric oxide (NO) from TG neurons helps regulate inflammatory signaling. However, 5-HT depletion leads to unopposed CGRP and NO release, exacerbating pain and inflammation [23–25]**.** Additionally, anti-nociceptive 5-HT receptors (5-HT_1B_, 5-HT_1D_, and 5-HT_1F_), which are targeted in migraine treatment, are known to mediate cerebral vasoconstriction and inhibit the release of inflammatory cytokines, including CGRP, NO, and substance P. A deficiency in 5-HT undermines this protective mechanism, resulting in elevated levels of inflammatory cytokines and cerebral vasodilation [4,26]. However, the positive correlations between RMP and total spikes, as well as between rheobase and AP rising time observed in the control-SM group, disappear following 5-HT depletion. This phenomenon may be attributed to the increased excitability of all neurons, effectively mimicking a state of suprathreshold stimulation. Taken together, these findings highlight the central role of 5-HT depletion in promoting peripheral and central sensitization through the increased intrinsic electrophysiological properties of the first-order SM and L-sized nociceptive TG neurons, thereby amplifying pain signaling in migraine.

Considering the size and nociceptive properties of first-order TG neurons, SM-sized TG neurons are predominantly nociceptive [15,16], whereas L-sized TG neurons serve dual roles, functioning as both nociceptive and non-nociceptive neurons [17]. These roles can be determined based on specific action potential AP properties. Previous studies have demonstrated that nociceptive L-sized neurons exhibit a wider AP duration, and reduced AP conduction velocity compared to their non-nociceptive counterparts [18–20]. Additionally, following stimulation by mediators such as 5-HT and acetylcholine, Vaden’s research demonstrated that type IIIa and IIIb neurons—classified as nociceptive large sized TG neurons—exhibited significantly higher peak amplitudes compared to type I, IIa, and IIb neurons, which are categorized as non-nociceptive neurons [20]. Furthermore, 5-HT depletion, particularly when combined with CSD, has been reported to disrupt the balance between nociceptive and somatosensory signals by shifting the AP properties of L-sized TG neurons toward a more nociceptive profile [6]. Consistent with our study, following 5-HT depletion induced by PCPA injection, significant negative correlations emerged in L-sized neurons between excitability parameters—specifically rheobase and threshold potential—and AP rising time. The observed increase in excitability, as indicated by reductions in rheobase and threshold potential, along with the prolongation of AP rising time which results in the AP widening and reduced upstroke velocity, suggests a transition of L-sized non-nociceptive neurons toward a nociceptive phenotype. The myelinated Ah-type neurons, a subset of myelinated neurons with electrophysiological properties intermediate between A-type myelinated neurons and C-type unmyelinated neurons, have recently garnered significant attention in research. These Ah neurons are known to play a critical role in nociception, especially in female rats, due to their gender-specific distribution and involvement in pain pathways [21,22]. However, in this study, we focused exclusively on male Wistar rats to minimize the influence of estrous cycle fluctuations on pain signal transmission [35]. Consequently, the contribution of Ah-type neurons to serotonin depletion–induced modulation of neuronal excitability could not be assessed. While Ah-type neurons have been predominantly described in female rats, it remains unclear whether comparable subtypes exist or contribute to nociceptive processing in males. Our findings indicate that 5-HT depletion drives a shift of L-sized neurons toward a more nociceptive-like electrophysiological profile. Given that Ah-type neurons are predominantly characterized in female models, their involvement cannot be determined in the present study and remains an important direction for future investigation.

Interestingly, after PCPA administration, several previously observed correlations were no longer evident. Notably, the strong positive correlation between total spikes and AP duration parameters, such as AP duration and AP falling time, which was observed in the control-L group, disappeared. Similarly, the positive correlations between AP overshoot and total spikes, as well as between AP overshoot and AP duration parameters, were also lost. In agreement with earlier findings, the results indicate that under normal conditions, L-sized neurons with wider AP durations could generate a higher number of AP spikes, reflecting their nociceptive tendency [18–20]. In addition to AP duration, AP amplitude—represented by AP overshoot, a key positive amplitude component—also correlates with the neuronal nociceptive tendency [20]. This association is highlighted by the correlations between AP overshoot and other parameters, including total spikes, AP falling, and AP duration, demonstrating that TG neurons with greater AP overshoot exhibit a heightened nociceptive tendency, as reflected by an increased number of total spikes and extended AP duration parameters. However, these correlations disappeared following 5-HT depletion, indicating that 5-HT depletion disrupts the normal AP-mediated nociceptive relationship association between total spikes and AP parameters (AP duration and AP overshoot) in L-sized neurons. This disruption could be attributed to an increased pain susceptibility across all L-sized neurons, regardless of their initial classification as nociceptive or non-nociceptive. In conclusion, the increased neuronal susceptibility in L-sized neurons may result from alterations in their properties toward a nociceptive trend, as well as a disruption in the equilibrium between nociceptive and non-nociceptive neurons.

Correlation analysis revealed negative correlations between total spikes and rheobase in both the PCPA-SM and PCPA-L groups with the PCPA-SM group demonstrated a stronger degree of correlation. This suggests that 5-HT depletion primarily increases the intrinsic electrophysiological properties of SM and L-sized nociceptive TG neurons, while having minimal or possibly inhibitory effects on L-sized non-nociceptive neurons. Apart from that, Kruskal–Wallis test tests revealed significant differences in total spikes, RMP and rheobase between the PCPA-SM and PCPA-L groups. The amplification of excitability and intrinsic electrophysiological alterations differences between these two neuron sizes may promote the process of sensitization, as explained by the gate control theory of pain. According to this theory, nociceptive signals are typically transmitted through non-myelinated neurons alongside inhibitory signals from myelinated Aβ neurons. In our experiment, under 5-HT depleted conditions, the relative difference in excitability changes between SM sized nociceptive and L-sized non-nociceptive neurons may cause the “opening” of the gate in the gate control theory of pain, facilitating the transmission of nociceptive signals from TG neurons [40].

In addition to the direct effects of 5-HT depletion on the intrinsic electrophysiological properties of TG neurons, the distribution and function of 5-HT receptor subtypes may further shape nociceptive processing within the trigeminovascular system. The 5-HT receptor family comprises seven major subgroups (5-HT₁–5-HT₇), each exhibiting distinct, and sometimes opposing roles in nociception. While traditionally classified as pro- or anti-nociceptive [26], emerging evidence suggests that receptor function is highly context-dependent, particularly with respect to anatomical localization. For example, 5-HT_3_ receptors illustrate this location-dependent duality [27,41]. At peripheral terminals of TG neurons, their activation enhances neuronal excitability and promotes CGRP release, thereby facilitating peripheral sensitization. In contrast, centrally located 5-HT_3_ receptors may exert inhibitory effects through presynaptic modulation. Based on prior evidence and the present findings, we propose a conceptual framework in which serotonin availability dynamically regulates the balance between pro- and anti-nociceptive receptor signaling. Under conditions of adequate 5-HT, the inhibitory influence of 5-HT_1B/1D/1F_ receptors may be functionally counteracted by concurrent activation of excitatory pathways. In contrast, during 5-HT depletion, reduced activation of these inhibitory receptors may shift the system toward a net pro-nociceptive state by enhancing neuronal excitability. At the central level, diminished serotonergic signaling may further weaken inhibitory control, thereby facilitating central sensitization. Accordingly, these interpretations should be considered as hypothesis-generating and will require direct experimental validation.

Linking these findings to clinical symptoms, the heightened intrinsic electrophysiological alterations accelerates the peripheral sensitization of nociceptive SM-sized and L-sized TG neurons, intensifying pain perception and contributing to hyperalgesia. Additionally, the sensitization of L-sized neurons disrupts the intricate balance between pain and somatosensory processing by altering their nociceptive properties, leading to abnormal somatosensation, such as cutaneous allodynia. This disruption further fuels central sensitization, ultimately driving migraine chronification. Thus, 5-HT depletion may serve as a key factor in increasing the susceptibility to migrainous pain and dysfunctional somatosensory processing by promoting both peripheral and central sensitization within the trigeminovascular system. Beyond migraine, serotonin dysregulation has also been implicated in other chronic pain conditions, such as irritable bowel syndrome and fibromyalgia, which involve similar sensitization processes [42,43]. The use of naïve TG neurons represents a reductionist approach that enables isolation of the direct effects of serotonergic depletion on intrinsic neuronal excitability, without confounding influences from migraine-related or inflammatory conditions. In this context, the observed changes should be interpreted as a potential priming mechanism that may enhanced neuronal excitability associated with migraine susceptibility, rather than a direct model of migraine pathology.

This study has several limitations. First, we focused on the intrinsic electrophysiological properties of trigeminal ganglion TG neurons without investigating underlying molecular mechanisms, including specific 5-HT receptor involvement and downstream inflammatory mediators such as CGRP, nitric oxide (NO), and adhesion molecules (ICAM/VCAM). Second, our analysis was limited to baseline neuronal susceptibility following 5-HT depletion and did not incorporate migraine-related stimuli, such as CGRP. Therefore, the interaction between 5-HT depletion and migraine-related triggers remains to be determined. Third, although serotonin depletion was induced using a well-established PCPA protocol, direct measurement of 5-HT levels, particularly within TG tissue, was not performed. Finally, only male rats were included to minimize hormonal variability, which may limit generalizability, especially given known sex differences and the presence of female-prevalent Ah-type neurons. Despite these limitations, the present findings provide direct functional evidence of altered neuronal excitability at the cellular level, which may represent a fundamental component of trigeminovascular sensitization under serotonin-depleted conditions.

In future studies, we aim to integrate ionic current analysis with biomolecular investigations—including inflammatory mediators and 5-HT receptor profiling—to further elucidate the molecular mechanisms of nociceptive signaling and receptor interactions within the trigeminovascular pathway. We also plan to incorporate migraine-relevant experimental paradigms, including established in vivo migraine models and CGRP stimulation, to determine how 5-HT depletion modulates neuronal responsiveness under pathophysiological conditions. Importantly, such approaches will allow direct testing of whether the heightened intrinsic excitability observed in the present study may translate into enhanced susceptibility to migraine-like nociceptive signaling. Additionally, future studies will incorporate female models to address the well-established sex differences in migraine pathophysiology and to determine whether the effects of serotonin depletion on trigeminal ganglion neuron excitability are sex-dependent [35,38]. In particular, these investigations will enable direct evaluation of the potential contribution of Ah-type nociceptive neurons, which have been reported to be more prevalent in females, under conditions of altered serotonergic tone, thereby further extending the physiological and translational relevance of our findings.

Our findings reveal that 5-HT depletion reshapes the intrinsic electrophysiological landscape of first-order TG neurons, promoting a state of heightened neuronal excitability that may predispose these cells to enhanced nociceptive signaling. This shift in baseline neuronal behavior provides a plausible mechanistic link between serotonergic imbalance and peripheral sensitization, a key feature of migraine pathophysiology and may contribute to clinically relevant phenomena such as hyperalgesia and cutaneous allodynia. While our data are confined to electrophysiological observations, they offer a foundation for understanding how disrupted serotonergic tone may prime the trigeminovascular system for exaggerated responses to migraine-related stimuli. Further studies will be required to validate these mechanisms and define their broader physiological and clinical relevance. These cellular-level alterations may provide a mechanistic framework bridging serotonergic dysregulation with aberrant nociceptive processing in migraine.

## Supporting information

S1 Table Correlation analysis in the (A) Control-SM and (B) PCPA-SM Groups. Pearson correlation coefficients (r) and corresponding *p*-values for all pairwise parameter correlations in the Control-SM and PCPA-SM groups. Raw data underlying Fig 1A and 1B. * Correlation is significant at the 0.05 level (2-tailed), **Correlation is significant at the 0.01 level (2-tailed).
(PDF)

S2 Table Correlation analysis in the (A) Control-L and (B) PCPA-L Groups. Pearson correlation coefficients (r) and corresponding p-values for all pairwise parameter correlations in the Control-L and PCPA-L groups. Raw data underlying Fig 2A and 2B. * Correlation is significant at the 0.05 level (2-tailed), **Correlation is significant at the 0.01 level (2-tailed).
(PDF)

S3 Table Electrophysiological parameters of trigeminal ganglion neurons. Electrophysiological parameters of trigeminal ganglion neurons in the control-SM, control-L, PCPA-SM, and PCPA-L groups. p^#^ indicates the pairwise comparison between the PCPA-SM and PCPA-L groups. No statistically significant differences were observed in the remaining pairwise comparisons. Raw data for Fig 3.
(PDF)
