# Supplementary material for: Serotonin depletion amplifies migraine susceptibility through trigeminal ganglion neurons sensitization: Insights from patch clamp recordings in rat model
Source: PLoS One. 2026 Aug 6;21(8):e0354679. doi: 10.1371/journal.pone.0354679 (PMC13446679; doi:10.1371/journal.pone.0354679)
Supplement: Supplementary file 1 [file pone.0354679.s001.pdf]

S1 Table (A)

		RMP	rheobase	threshold	totalspike	APheight	APovershoot	APrising	APfalling	APduration
RMP	Pearson Correlation	1	.026	.295	.560*	.464	.315	-.162	-.212	-.246
	Sig. (2-tailed)		.933	.327	.047	.110	.295	.597	.487	.418
rheobase	Pearson Correlation	.026	1	.215	-.525	.040	.457	-.605*	.059	-.302
	Sig. (2-tailed)	.933		.481	.065	.896	.116	.028	.849	.316
threshold	Pearson Correlation	.295	.215	1	.023	.043	.087	-.075	.007	-.038
	Sig. (2-tailed)	.327	.481		.942	.890	.777	.808	.982	.903
totalspike	Pearson Correlation	.560*	-.525	.023	1	.479	.082	.295	-.164	.048
	Sig. (2-tailed)	.047	.065	.942		.098	.789	.329	.591	.876
APheight	Pearson Correlation	.464	.040	.043	.479	1	.835**	.132	.284	.282
	Sig. (2-tailed)	.110	.896	.890	.098		.000	.667	.347	.351
APovershoot	Pearson Correlation	.315	.457	.087	.082	.835**	1	-.184	.361	.158
	Sig. (2-tailed)	.295	.116	.777	.789	.000		.547	.225	.607
APrising	Pearson Correlation	-.162	-.605*	-.075	.295	.132	-.184	1	.178	.699**
	Sig. (2-tailed)	.597	.028	.808	.329	.667	.547		.561	.008
APfalling	Pearson Correlation	-.212	.059	.007	-.164	.284	.361	.178	1	.828**
	Sig. (2-tailed)	.487	.849	.982	.591	.347	.225	.561		.000
APduration	Pearson Correlation	-.246	-.302	-.038	.048	.282	.158	.699**	.828**	1
	Sig. (2-tailed)	.418	.316	.903	.876	.351	.607	.008	.000	

S1 Table (B)

		RMP	rheobase	threshold	totalspike	APheight	APovershoot	APrising	APfalling	APduration
RMP	Pearson Correlation	1	-.146	.037	.152	.030	-.435	-.145	.554	.164
	Sig. (2-tailed)		.668	.913	.656	.931	.181	.669	.077	.630
rheobase	Pearson Correlation	-.146	1	.735**	-.726*	.046	.302	-.198	-.063	-.183
	Sig. (2-tailed)	.668		.010	.011	.893	.367	.559	.855	.590
threshold	Pearson Correlation	.037	.735**	1	-.812**	.065	.217	-.344	.293	-.118
	Sig. (2-tailed)	.913	.010		.002	.850	.521	.301	.383	.730
totalspike	Pearson Correlation	.152	-.726*	-.812**	1	-.065	-.173	-.037	-.213	-.134
	Sig. (2-tailed)	.656	.011	.002		.850	.611	.913	.530	.693
APheight	Pearson Correlation	.030	.046	.065	-.065	1	.673*	.622*	.546	.748**
	Sig. (2-tailed)	.931	.893	.850	.850		.023	.041	.082	.008
APovershoot	Pearson Correlation	-.435	.302	.217	-.173	.673*	1	.272	.261	.338
	Sig. (2-tailed)	.181	.367	.521	.611	.023		.419	.438	.309
APrising	Pearson Correlation	-.145	-.198	-.344	-.037	.622*	.272	1	.217	.874**
	Sig. (2-tailed)	.669	.559	.301	.913	.041	.419		.522	.000
APfalling	Pearson Correlation	.554	-.063	.293	-.213	.546	.261	.217	1	.664*
	Sig. (2-tailed)	.077	.855	.383	.530	.082	.438	.522		.026
APduration	Pearson Correlation	.164	-.183	-.118	-.134	.748**	.338	.874**	.664*	1
	Sig. (2-tailed)	.630	.590	.730	.693	.008	.309	.000	.026	
