# Supplementary material for: Serotonin depletion amplifies migraine susceptibility through trigeminal ganglion neurons sensitization: Insights from patch clamp recordings in rat model
Source: PLoS One. 2026 Aug 6;21(8):e0354679. doi: 10.1371/journal.pone.0354679 (PMC13446679; doi:10.1371/journal.pone.0354679)
Supplement: Supplementary file 2 [file pone.0354679.s002.pdf]

S2 Table (A)

		RMP	rheobase	threshold	totalspike	APheight	APovershoot	APrising	APfalling	APduration
RMP	Pearson Correlation	1	.423	.169	-.065	.089	.104	-.175	.017	-.054
	Sig. (2-tailed)		.171	.599	.840	.784	.747	.585	.958	.867
rheobase	Pearson Correlation	.423	1	-.242	-.358	.256	-.005	-.507	-.210	-.365
	Sig. (2-tailed)	.171		.449	.254	.421	.988	.093	.512	.244
threshold	Pearson Correlation	.169	-.242	1	-.311	-.311	-.513	-.284	-.347	-.388
	Sig. (2-tailed)	.599	.449		.326	.325	.088	.371	.269	.212
totalspike	Pearson Correlation	-.065	-.358	-.311	1	-.138	.817**	.418	.949**	.923**
	Sig. (2-tailed)	.840	.254	.326		.670	.001	.177	.000	.000
APheight	Pearson Correlation	.089	.256	-.311	-.138	1	.376	.292	-.019	.098
	Sig. (2-tailed)	.784	.421	.325	.670		.228	.356	.953	.763
APovershoot	Pearson Correlation	.104	-.005	-.513	.817**	.376	1	.333	.852**	.813**
	Sig. (2-tailed)	.747	.988	.088	.001	.228		.290	.000	.001
APrising	Pearson Correlation	-.175	-.507	-.284	.418	.292	.333	1	.334	.654*
	Sig. (2-tailed)	.585	.093	.371	.177	.356	.290		.289	.021
APfalling	Pearson Correlation	.017	-.210	-.347	.949**	-.019	.852**	.334	1	.931**
	Sig. (2-tailed)	.958	.512	.269	.000	.953	.000	.289		.000
APduration	Pearson Correlation	-.054	-.365	-.388	.923**	.098	.813**	.654*	.931**	1
	Sig. (2-tailed)	.867	.244	.212	.000	.763	.001	.021	.000	

S2 Table (B)

		RMP	rheobase	threshold	totalspike	APheight	APovershoot	APrising	APfalling	APduration
RMP	Pearson Correlation	1	-.289	-.277	.094	.091	.152	.123	-.213	-.096
	Sig. (2-tailed)		.337	.360	.759	.767	.620	.688	.485	.756
rheobase	Pearson Correlation	-.289	1	.501	-.570*	.066	.184	-.694**	.251	-.212
	Sig. (2-tailed)	.337		.081	.042	.830	.548	.008	.408	.488
threshold	Pearson Correlation	-.277	.501	1	-.325	-.428	-.302	-.565*	.187	-.186
	Sig. (2-tailed)	.360	.081		.278	.144	.316	.044	.540	.544
totalspike	Pearson Correlation	.094	-.570*	-.325	1	.342	.233	.289	-.012	.161
	Sig. (2-tailed)	.759	.042	.278		.253	.444	.338	.969	.599
APheight	Pearson Correlation	.091	.066	-.428	.342	1	.968**	-.016	.291	.221
	Sig. (2-tailed)	.767	.830	.144	.253		.000	.958	.335	.469
APovershoot	Pearson Correlation	.152	.184	-.302	.233	.968**	1	-.134	.375	.218
	Sig. (2-tailed)	.620	.548	.316	.444	.000		.662	.206	.475
APrising	Pearson Correlation	.123	-.694**	-.565*	.289	-.016	-.134	1	.026	.611*
	Sig. (2-tailed)	.688	.008	.044	.338	.958	.662		.934	.026
APfalling	Pearson Correlation	-.213	.251	.187	-.012	.291	.375	.026	1	.807**
	Sig. (2-tailed)	.485	.408	.540	.969	.335	.206	.934		.001
APduration	Pearson Correlation	-.096	-.212	-.186	.161	.221	.218	.611*	.807**	1
	Sig. (2-tailed)	.756	.488	.544	.599	.469	.475	.026	.001	
