# Supplementary material for: Serotonin depletion amplifies migraine susceptibility through trigeminal ganglion neurons sensitization: Insights from patch clamp recordings in rat model
Source: PLoS One. 2026 Aug 6;21(8):e0354679. doi: 10.1371/journal.pone.0354679 (PMC13446679; doi:10.1371/journal.pone.0354679)
Supplement: Supplementary file 3 [file pone.0354679.s003.pdf]

Figure	Parameters	Control-SM (N=13)	Control-L (N=12)	PCPA-SM (N=11)	PCPA-L (N=13)	p-value Kruskal	p-value ANOVA
**Fig 3B**	RMP (mV)	-58.60±11.31	-63.89±10.59	-51.25±7.99	-61.36±8.21	0.012*	-
				$p^{\#} < 0.001$			
**Fig 3C**	Rheobase (pA)	13.85±10.24	21.25±17.60	13.64±11.20	40.77±23.26	0.003*	-
				$p^{\#} = 0.012$			
**Fig 3D**	Threshold (mV)	-42.39±8.38	-36.35±13.39	-40.02±7.05	-34.07±8.94	-	0.152
**Fig 3A**	Total Spikes	160.62±169.93	28.58±50.18	189.91±205.63	13.69 ±16.30	0.001*	-
				$p^{\#} = 0.004$			
**Fig 3H**	AP height (mV)	97.12±10.46	97.36±16.11	97.75±13.77	100.12±17.05		0.950
**Fig 3G**	AP overshoot (mV)	59.83±15.78	65.62±25.50	61.01±8.30	63.36±17.82	0.863	-
**Fig 3E**	AP rising (msec)	1.06±0.62	0.90±0.51	1.13±0.63	0.80±0.40	0.364	-
**Fig 3F**	AP falling (msec)	1.27±0.79	1.03±1.07	1.06±0.41	0.92±0.54	0.067	-
**Fig 3I**	AP duration (msec)	2.34±1.09	1.93±1.33	2.20±0.83	1.72±0.68	0.168	-
