# Supplementary material for: Serotonin depletion amplifies migraine susceptibility through trigeminal ganglion neurons sensitization: Insights from patch clamp recordings in rat model
Source: PLoS One. 2026 Aug 6;21(8):e0354679. doi: 10.1371/journal.pone.0354679 (PMC13446679; doi:10.1371/journal.pone.0354679)
Supplement: Supplementary file 4 [file pone.0354679.s005.docx]

**Response to reviewers**

**Reviewer #1**: Refine the Abstract and Introduction:

The abstract mentions the possibility of 5-HT depletion disturbing receptor balance. Given that this is a key part of the discussion, consider softening this or framing it more as a hypothesis generated by the data, as the study did not directly measure receptor function.

**Authors’ Response:**

We thank the reviewer for this important and insightful comment. We agree that the statement regarding disruption of 5-HT receptor balance should be interpreted with appropriate caution, as receptor-specific mechanisms were not directly assessed in this study.

Accordingly, we have revised the Abstract to more clearly frame this point as a hypothesis generated from our electrophysiological findings, rather than as a definitive mechanistic conclusion. This revision ensures that our interpretation remains consistent with the scope of the data while still highlighting a biologically plausible implication that may guide future investigations.

**Changes in manuscript:**

The relevant sentence in the Abstract has been revised to explicitly reflect a hypothesis-generating interpretation rather than a causal inference (page 2, lines 22–27).

In the introduction, the transition from general migraine susceptibility to the specific concept of "intrinsic neuronal susceptibility" could be slightly smoother. A sentence explicitly stating that this study aims to define this susceptibility at the cellular level in first-order neurons would strengthen the narrative.

**Authors’ Response:**

We appreciate the reviewer’s constructive suggestion. We agree that a clearer conceptual transition would improve the readability and logical flow of the Introduction.

In response, we have revised the relevant section to provide a more gradual progression from general migraine susceptibility to the specific concept of “intrinsic neuronal susceptibility.” Importantly, we have added a clarifying sentence explicitly stating that this study aims to define this susceptibility at the cellular level of first-order trigeminal ganglion neurons. This addition strengthens the conceptual framework and more clearly positions our study within the broader context of migraine pathophysiology.

**Changes in manuscript:**

A new sentence has been added to the Introduction to explicitly define the study objective and improve conceptual continuity (page 4–5, lines 45–77; page 7, lines 134–143).

Clarify the Discussion:
The discussion is comprehensive but somewhat lengthy. It could be more focused. The section on other disorders (IBS, fibromyalgia) is interesting but somewhat tangential. Consider condensing this to maintain a sharp focus on migraine mechanisms.

**Authors’ Response**:

We thank the reviewer for this constructive and helpful suggestion. We agree that improving the focus and conciseness of the Discussion strengthens the overall clarity of the manuscript.

Accordingly, we have revised the Discussion to achieve a more streamlined and migraine-centered narrative. In particular, the section addressing other serotonin-related disorders, including irritable bowel syndrome (IBS) and fibromyalgia, has been substantially condensed. While these conditions remain briefly mentioned to contextualize the broader relevance of serotonergic dysregulation, excessive detail has been removed to maintain a sharper focus on migraine-specific mechanisms.

These revisions improve readability while preserving the conceptual contribution of our findings within the context of migraine pathophysiology.

**Changes in manuscript:**

The Discussion section has been condensed, with the section on IBS and fibromyalgia substantially shortened and refocused (page 27, lines 536–543).

The argument about 5-HT receptor hierarchy (page 28-29) is insightful but speculative. It would be beneficial to explicitly state that this is a proposed model based on the literature and the current findings, and that it requires direct experimental validation.

**Authors’ Response:**

We appreciate the reviewer’s insightful comment. We agree that the proposed 5-HT receptor hierarchy represents a conceptual interpretation rather than a directly tested mechanism in the present study.

In response, we have revised the relevant section to explicitly frame this as a hypothesis-driven model derived from existing literature in combination with our electrophysiological findings. We have also clearly stated that this model requires direct experimental validation, particularly through receptor-specific and molecular investigations.

This clarification ensures appropriate distinction between data-driven conclusions and broader mechanistic interpretation, thereby improving the scientific rigor and transparency of the Discussion.

**Changes in manuscript:**

The section discussing 5-HT receptor hierarchy has been revised to explicitly identify it as a proposed model and to highlight the need for direct experimental validation (page 25, lines 487–505).

Address Limitations More Proactively:
The limitation of using only male rats is well-noted. However, when discussing the Ah-type neurons (which are female-prevalent) and their potential role, the language should be more cautious. The current text (Page 27, lines 381-383) presents a hypothesis that the study's design cannot address. Frame this more clearly as a compelling question for future research directly in female models.

**Authors’ Response**:

We thank the reviewer for this important and insightful comment. We fully agree that the discussion of Ah-type neurons requires more cautious interpretation, particularly given the male-only design of the present study.

Accordingly, we have revised the text to clearly acknowledge that, although our findings suggest that large-sized TG neurons may shift toward a more nociceptive-like electrophysiological profile following 5-HT depletion, the specific involvement of Ah-type neurons—predominantly characterized in female models—cannot be determined from our data.

This point has now been explicitly reframed as a key question for future investigation. We emphasize the importance of studies in female models to directly assess the role of Ah-type neurons and to better understand sex-specific mechanisms in migraine pathophysiology.

These revisions ensure that our interpretation remains aligned with the experimental scope while highlighting a meaningful direction for future research.

**Changes in manuscript:**

The relevant section has been revised to adopt a more cautious interpretation and to reposition the role of Ah-type neurons as a question for future studies in female models (page 23, lines 444–451).

Minor Technical Points:

Figure 3: Ensure the figure legend clearly states which statistical test was used for each panel (Kruskal-Wallis vs. ANOVA) and that the pairwise comparisons shown are post-hoc tests following a significant omnibus test.

**Authors’ response**:

We thank the reviewer for this helpful and important suggestion. We agree that clear reporting of statistical methods in figure legends is essential for transparency and reproducibility.

Accordingly, we have revised the Figure 3 legend to explicitly specify the statistical test applied to each parameter, indicating whether a Kruskal–Wallis test or one-way ANOVA was used based on data distribution. We have also clarified that the pairwise comparisons shown in the figure represent post-hoc analyses performed only following a significant omnibus test.

These revisions improve the clarity and interpretability of the statistical presentation.

**Changes in manuscript:**

The Figure 3 legend has been revised to explicitly describe the statistical tests used for each panel and the conditions under which post-hoc comparisons were conducted (page 19, lines 364–367). 

Results Flow: The results section jumps between correlation analysis and group comparisons. A brief introductory sentence for each sub-section (e.g., "We first sought to understand the relationship between...") could improve readability.

**Authors’ response**:

We appreciate the reviewer’s insightful suggestion. We agree that improving the structural organization of the Results section enhances readability and comprehension.

In response, we have reorganized the Results into clearly defined subsections that separate correlation analyses from intergroup comparisons. Additionally, brief introductory sentences have been incorporated at the beginning of each subsection to clarify the analytical objective and guide the reader through the progression of the results.

These revisions improve the logical flow and overall clarity of the Results section.

**Changes in manuscript:**

The Results section has been restructured into subsections with added introductory statements to improve clarity and narrative flow (page 12, lines 252–254; page 13, lines 276–278; page 15, lines 303–306; page 16, lines 333–335).

**Reviewer #2**: The study by Saknan et al addressed the influence of serotonin depletion on the excitability of TG neurons. Althougt this is a relevant issue, several flaws reder the manuscript not aceptable for publication in its current form.The major points raised during my revision are:

1- The introduction is too long.

**Authors’ response**:

We thank the reviewer for this constructive comment. We agree that the original Introduction was overly detailed, which may have affected clarity and focus.

In response, we have substantially revised and streamlined the Introduction to improve conciseness and readability. Redundant background information has been removed, and the section has been reorganized to more clearly emphasize the key knowledge gap and the rationale for the present study. In particular, we have tightened the narrative to focus more directly on the role of serotonin depletion in trigeminal ganglion neuronal excitability and its relevance to migraine pathophysiology.

As a result of these revisions, the length of the Introduction has been reduced from 862 to 654 words (approximately a 24% reduction), leading to a more focused and accessible presentation.

These revisions result in a more focused and accessible Introduction that better guides the reader to the objectives of the study.

**Changes in manuscript:**

The Introduction has been substantially condensed and reorganized to improve clarity, focus, and narrative flow (page 3, lines 45–77; page 7, lines 134–143).

2- Although some parts of the methods section are very detailed, some information is missing.For example, how long after 5HT depletion the TGs were collected for culture? How long after the culture, the authors performed patch clamp?

**Authors’ response**:

We thank the reviewer for this important and insightful comment. We agree that precise timing of experimental procedures is essential for clarity and reproducibility.

In response, we have revised the Methods section to explicitly clarify the temporal sequence of the experimental protocol. Specifically, 5-HT depletion was induced using a well-established PCPA regimen (100 mg/kg, i.p., for three consecutive days), which has been shown to produce substantial central and systemic serotonin depletion within 24 hours after the final injection [1].

In the present study, trigeminal ganglia were harvested 24 hours after the final PCPA administration, corresponding to a time point at which serotonin depletion is well established based on prior literature. Following enzymatic dissociation, TG neurons were cultured for 18–24 hours prior to electrophysiological recordings, and whole-cell patch-clamp experiments were performed within this defined time window.

These details have now been explicitly incorporated into the revised Methods section to improve transparency and reproducibility.

**Changes in manuscript:**

The Methods section has been revised to clearly specify the timing of TG harvesting and electrophysiological recordings following 5-HT depletion (page 9, lines 183–184). Additionally, we have added new reference #36 (page 36, lines 725–726).

3- There are no controls for serotonin depletion. How do the authors know that the protocol is working properly? How do they know that the depletion occurs at the TG level? How long the depletion lasts?

**Authors’ response**:

We thank the reviewer for this critical and important comment. We agree that validation of serotonin depletion is essential for the interpretation of our findings.

In the present study, 5-HT depletion was induced using a well-established PCPA protocol (100 mg/kg, i.p., for three consecutive days), which has been extensively validated across multiple studies to produce robust and reproducible depletion of serotonin in both central and peripheral compartments [1–3]. Specifically, this regimen has been shown to reduce brain 5-HT levels to approximately 10–20% of control within 24 hours after the final injection.

Importantly, PCPA inhibits tryptophan hydroxylase systemically, leading to global suppression of serotonin synthesis. Previous studies have demonstrated that this results not only in central depletion but also in marked reductions in circulating (whole blood and plasma) serotonin, as well as in peripheral tissues such as the spleen and gastrointestinal tract [1,4]. Given that trigeminal ganglion neurons are part of the peripheral nervous system and are directly exposed to systemic biochemical changes, this systemic depletion strongly supports a reduction in serotonergic tone at the TG level. Additional studies have also demonstrated profound central serotonin depletion under alternative dosing regimens [5].

Regarding the temporal profile, serotonin depletion develops progressively over the 2–3 day administration period and remains markedly reduced for at least 24 hours following the final injection [1–3]. In our study, TG tissues were collected at this validated time point (24 hours post-final dose), ensuring that electrophysiological recordings were performed during a period of established depletion.

We acknowledge that serotonin levels were not directly measured in the trigeminal ganglion in the present study. This represents a limitation, and we have now explicitly stated this in the revised manuscript. However, given the well-documented systemic and central effects of this PCPA protocol, we believe it provides strong and widely accepted indirect evidence supporting effective serotonin depletion during our experimental window.

**Changes in manuscript:**

We have clarified the rationale and temporal validity of the PCPA-induced serotonin depletion in the Methods section (page 8, lines 166–170) and have explicitly acknowledged the lack of direct TG-level measurement as a limitation in the Discussion (page 28, lines 565–567).

4- The conclusion is based solely in patch clamp experiments.

**Authors’ response**:

We thank the reviewer for this important comment. We agree that our conclusions are primarily derived from patch-clamp electrophysiological recordings of trigeminal ganglion neurons.

In response, we have revised both the Discussion and Conclusion sections to more clearly delineate the scope of our study. Specifically, we now emphasize that our findings pertain to intrinsic electrophysiological alterations in TG neurons following 5-HT depletion, without directly addressing underlying molecular mechanisms or in vivo functional outcomes.

Accordingly, statements that could be interpreted as overextending beyond the electrophysiological data have been carefully moderated. We have also explicitly acknowledged that additional molecular, biochemical, and in vivo studies are required to further validate these findings and to establish their translational relevance in migraine pathophysiology.

Importantly, we highlight that the present study provides a cellular-level functional framework that may help inform future mechanistic and translational investigations.

**Changes in manuscript:**

The Discussion and Conclusion sections have been revised to better align the interpretation of the findings with the electrophysiological scope of the study and to explicitly acknowledge the need for further validation.

5- The discussion is too speculative. The authors present several information about the influcence of serotonin depletion in general, but they did not investigate the influence of this depletion on the TG, besides the eletrophysiological properties of the neurons. In my opinion the data is too preliminary and the study requires further experiments to show a consistent idea.

**Authors’ response**:

We thank the reviewer for this thoughtful and important comment. We agree that our study is focused on the electrophysiological characterization of trigeminal ganglion neurons following 5-HT depletion and does not directly assess the associated molecular, biochemical, or in vivo mechanisms.

In response, we have carefully revised the Discussion to reduce speculative statements and to more clearly distinguish between our experimental findings and broader interpretations. In particular, we have moderated sections that extend beyond the electrophysiological data and explicitly framed these as hypotheses or conceptual interpretations rather than definitive conclusions.

We now emphasize that our findings demonstrate quantifiable alterations in intrinsic neuronal excitability and spike-generating properties, which provide functional evidence at the cellular level for increased neuronal susceptibility under 5-HT-depleted conditions. While these results do not establish direct mechanistic pathways, they offer a physiologically relevant framework that may underlie altered nociceptive processing within the trigeminovascular system.

We agree that further studies incorporating molecular, receptor-level, and in vivo approaches will be necessary to validate these interpretations and to establish causal links to migraine pathophysiology. This has now been explicitly acknowledged and highlighted in the revised manuscript.

**Changes in manuscript:**

The Discussion has been revised to reduce speculative language, clearly separate data-driven findings from hypothesis-generating interpretations, and to explicitly define the scope and limitations of the study (page 25, lines 487–505; page 30, lines 600–611).

6- The authors claim that this mechanism is relevant to migraine, but they performed all the recordings in the TG of naive rats. It would be very interesting to see the influence of 5HT depletion in TG from rats subjected to a migraine model or in TG cutured neurons treated with a migarine stimulus, such as CGRP.

**Authors’ response:**

We thank the reviewer for this insightful and important suggestion. We agree that incorporating established migraine models or applying migraine-related stimuli, such as CGRP, would further strengthen the translational relevance of our findings.

However, the primary aim of the present study was to define how serotonin depletion alters the intrinsic electrophysiological properties of trigeminal ganglion (TG) neurons under baseline conditions. By focusing on naïve TG neurons, we sought to isolate the direct impact of reduced serotonergic tone on neuronal excitability, independent of superimposed pathological or inflammatory stimuli.

Our results demonstrate that 5-HT depletion enhances intrinsic neuronal excitability, suggesting a shift toward a hyperresponsive state. We propose that this altered baseline excitability may represent a permissive or priming condition that increases the susceptibility of TG neurons to migraine-related triggers, including CGRP and inflammatory mediators, rather than directly modeling migraine pathology itself.

**Changes in manuscript:**

We acknowledge that the absence of a migraine model represents a limitation of the current study. This point has now been explicitly clarified in the revised Introduction (page 7, lines 134–140) and Discussion (page 21, lines 536–543), where we also highlight the need for future studies combining 5-HT depletion with migraine-relevant stimuli or in vivo models to establish causal links to migraine pathophysiology (page 29, lines 584–589).

7- In my opinion to investigate sex differences is crucial in migraine studies, since we can have very different results depending on the sex.

**Authors’ response**:

We thank the reviewer for this important and well-recognized point. We fully agree that sex differences are critically important in migraine research, given the well-established sex-specific variations in prevalence, hormonal modulation, and nociceptive processing.

In the present study, experiments were conducted in male rats to minimize variability associated with hormonal fluctuations and to allow clearer interpretation of the intrinsic electrophysiological effects of serotonin depletion. However, we acknowledge that this design limits the generalizability of our findings, particularly in the context of migraine, which disproportionately affects females.

**Changes in manuscript:**

This limitation has now been explicitly addressed in the revised Discussion (page 29, lines 589–595). Importantly, we highlight the need for future studies in female models to determine whether serotonin depletion exerts sex-specific effects on trigeminal ganglion neuron excitability, including the potential involvement of Ah-type neurons, which have been reported to be more prevalent in females.

**References**

1.	Koe BK, Weissman A. p-Chlorophenylalanine: a specific depletor of brain serotonin. J Pharmacol Exp Ther. 1966;154(3):499-516.

2.	Cao F, Leung LS. Effect of atropine and PCPA on the behavioral modulation of paired-pulse response in the hippocampal CA1 region. Brain Res. 1992;576(2):339-42. <https://doi.org/10.1016/0006-8993(92)90701-a>

3.	Supornsilpchai W, Sanguanrangsirikul S, Maneesri S, Srikiatkhachorn A. Serotonin depletion, cortical spreading depression, and trigeminal nociception. Headache. 2006;46(1):34-9. <https://doi.org/10.1111/j.1526-4610.2006.00310.x>

4.	Artigas F, Sarrias MJ, Martínez E, Gelpí E. Serotonin in body fluids: characterization of human plasmatic and cerebrospinal fluid pools by means of a new HPLC method. Life Sci. 1985;37(5):441-7. <https://doi.org/10.1016/0024-3205(85)90406-0>

5.	Huang G-J, Herbert J. Serotonin Modulates the Suppressive Effects of Corticosterone on Proliferating Progenitor Cells in the Dentate Gyrus of the Hippocampus in the Adult Rat. Neuropsychopharmacology. 2005;30(2):231-41. <https://doi.org/10.1038/sj.npp.1300609>
