# Supplementary material for: Serotonin depletion amplifies migraine susceptibility through trigeminal ganglion neurons sensitization: Insights from patch clamp recordings in rat model
Source: PLoS One. 2026 Aug 6;21(8):e0354679. doi: 10.1371/journal.pone.0354679 (PMC13446679; doi:10.1371/journal.pone.0354679)
Supplement: Supplementary file 5 [file pone.0354679.s006.docx]

**Response to reviewers**

**Reviewer #1**:
Redundancy and length – The Introduction and Discussion still contain some repetitive passages (e.g., the concept of "intrinsic neuronal susceptibility" is defined multiple times). Consider a final pruning for conciseness.

**Authors’ Response:**

We thank the reviewer for this helpful suggestion. We carefully reviewed the manuscript and further improved its conciseness by removing repetitive wording, particularly where the concept of intrinsic neuronal susceptibility was repeatedly described. In several instances, we replaced repetitive expressions with more precise terminology (e.g., neuronal hyperexcitability, intrinsic electrophysiological alterations, and altered baseline excitability) while preserving the intended scientific meaning.

**Changes in manuscript:**

The relevant wordings in the text been revised (Introduction: page 5, lines 73–74; page 6, lines 83–86, Discussion: page 17, lines 311–312; page 18, lines 332–333; page 19, lines 359–360; page 21, lines 415–416; page 21, lines 420; page 23, lines 446–447; page 23, lines 460–462).

Minor error in Table 1 – The footnote "p-value Pearson's Chi- square test" is incomplete; specify it is for bursting profiles.

**Authors’ Response:**

We thank the reviewer for this helpful comment. We have revised the Table 1 footnote to clarify that the reported Pearson's Chi-square test was used to compare the distribution of bursting profiles (single-spike vs. multiple-spike neurons) between groups, thereby improving the clarity and completeness of the statistical description.

**Changes in manuscript:**

The relevant sentence in the footnote Table 1 has been revised (page 11, lines 213–215).

References – The citation numbers in the text appear to have shifted; ensure consistency with the reference list.

**Authors’ Response:**

We thank the reviewer for this careful observation. We have thoroughly checked all in-text citations and verified their consistency with the revised reference list. Any numbering inconsistencies introduced during the previous revision have now been corrected.

Figure legends – Figure 3 legend correctly specifies Kruskal-Wallis vs ANOVA, but ensure the figure panels (A–I) are clearly labeled in the submitted figure file.

**Authors’ Response:**

We thank the reviewer for this helpful comment. We have carefully checked the submitted Figure 3 file and ensured that all panels (A–I) are clearly labeled and correspond consistently with the descriptions provided in the figure legend.

**Changes in manuscript:**

The relevant sentence in the figure legends of Figure 3 has been revised (page 16, lines 305–308).

Language – A few awkward phrasing remain (e.g., "the experiment raises the possibility that 5-HT depletion may also indirectly disturb" in abstract is repetitive). A light copyedit would help.

**Authors’ Response:**

We thank the reviewer for this helpful comment. The manuscript has undergone an additional round of careful language editing to improve clarity, conciseness, and readability. We have also corrected several awkward or repetitive phrases throughout the manuscript, including in the Abstract, Introduction, and Discussion.

Overall, the scientific content is sound, the methodology is appropriate, and the conclusions are now appropriately tempered. The study provides a useful electrophysiological foundation for understanding serotonergic modulation of trigeminal ganglion neurons.

**Authors’ Response:**

We sincerely thank the reviewer for the careful evaluation of our manuscript throughout the review process and for this encouraging final assessment. We greatly appreciate the reviewer’s constructive comments, which have helped us substantially improve the clarity, rigor, and balance of the manuscript. We are pleased that the revised version is now considered scientifically sound and that our study provides a useful electrophysiological foundation for understanding serotonergic modulation of trigeminal ganglion neurons.
