# Supplementary material for: Serotonin depletion amplifies migraine susceptibility through trigeminal ganglion neurons sensitization: Insights from patch clamp recordings in rat model
Source: PLoS One. 2026 Aug 6;21(8):e0354679. doi: 10.1371/journal.pone.0354679 (PMC13446679; doi:10.1371/journal.pone.0354679)
Supplement: Peer review history

# Peer review: Decision Letter 0

**DOI:** 10.1371/journal.pone.0354679.r001

**Authors:** Paulo Ho

9 Apr 2026

PONE-D-25-46193

Serotonin depletion amplifies migraine susceptibility through trigeminal ganglion neurons sensitization: Insights from patch clamp recordings in rat model

PLOS One

Dear Dr. Bongsebandhu-phubhakdi,

Thank you for submitting your manuscript to PLOS ONE. After careful consideration, we feel that it has merit but does not fully meet PLOS ONE’s publication criteria as it currently stands. Therefore, we invite you to submit a revised version of the manuscript that addresses the points raised during the review process, in special:

1)  How the serotonin depletion was controlled in the experiments;

2) The claim that this mechanism is irrelevant to migraine was based on recordings in the TG of naive rats. It would be interesting to see the influence of 5HT depletion in TG from rats subjected to a migraine model or TG cultured neurons treated with a migraine stimulus like CGRP, for instance, as suggested by reviewer #2. Or at least, included in the discussion;

3) Please, answer all the comments raised by both the reviewers.

Please submit your revised manuscript by May 24 2026 11:59PM. If you will need more time than this to complete your revisions, please reply to this message or contact the journal office at plosone@plos.org. When you're ready to submit your revision, log on to https://www.editorialmanager.com/pone/ and select the 'Submissions Needing Revision' folder to locate your manuscript file.

Please include the following items when submitting your revised manuscript:

- A letter that responds to each point raised by the academic editor and reviewer(s). You should upload this letter as a separate file labeled 'Response to Reviewers'.
- A marked-up copy of your manuscript that highlights changes made to the original version. You should upload this as a separate file labeled 'Revised Manuscript with Track Changes'.
- An unmarked version of your revised paper without tracked changes. You should upload this as a separate file labeled 'Manuscript'.

If you would like to make changes to your financial disclosure, please include your updated statement in your cover letter. Guidelines for resubmitting your figure files are available below the reviewer comments at the end of this letter.

If applicable, we recommend that you deposit your laboratory protocols in protocols.io to enhance the reproducibility of your results. Protocols.io assigns your protocol its own identifier (DOI) so that it can be cited independently in the future. For instructions see: https://journals.plos.org/plosone/s/submission-guidelines#loc-laboratory-protocols. Additionally, PLOS ONE offers an option for publishing peer-reviewed Lab Protocol articles, which describe protocols hosted on protocols.io. Read more information on sharing protocols at https://plos.org/protocols?utm_medium=editorial-email&utm_source=authorletters&utm_campaign=protocols.

As the corresponding author, your ORCID iD is verified in the submission system and will appear in the published article. PLOS supports the use of ORCID, and we encourage all coauthors to register for an ORCID iD and use it as well. Please encourage your coauthors to verify their ORCID iD within the submission system before final acceptance, as unverified ORCID iDs will not appear in the published article. *Only*  the individual author can complete the verification step; PLOS staff *cannot*  verify ORCID iDs on behalf of authors.

We look forward to receiving your revised manuscript.

Kind regards,

Paulo Lee Ho, Ph.D.

Academic Editor

PLOS One

Journal Requirements:

When submitting your revision, we need you to address these additional requirements.

1. Please ensure that your manuscript meets PLOS ONE's style requirements, including those for file naming. The PLOS ONE style templates can be found at

https://journals.plos.org/plosone/s/file?id=wjVg/PLOSOne_formatting_sample_main_body.pdf and

https://journals.plos.org/plosone/s/file?id=ba62/PLOSOne_formatting_sample_title_authors_affiliations.pdf

2. Thank you for stating the following financial disclosure: [his research is funded by Thailand Science and Research Innovation Fund, Chulalongkorn University- Fundamental Fund (HEA_FF_68_275_3000_041) and Quick Win research project, Ratchadapiseksompotch Fund, Chulalongkorn University (No.767006).].

Please state what role the funders took in the study.  If the funders had no role, please state: ""The funders had no role in study design, data collection and analysis, decision to publish, or preparation of the manuscript.""

If this statement is not correct you must amend it as needed.

Please include this amended Role of Funder statement in your cover letter; we will change the online submission form on your behalf.

3. We note that your Data Availability Statement is currently as follows: [All relevant data are within the manuscript and its Supporting Information files.]

Please confirm at this time whether or not your submission contains all raw data required to replicate the results of your study. Authors must share the “minimal data set” for their submission. PLOS defines the minimal data set to consist of the data required to replicate all study findings reported in the article, as well as related metadata and methods (https://journals.plos.org/plosone/s/data-availability#loc-minimal-data-set-definition).

For example, authors should submit the following data:

- The values behind the means, standard deviations and other measures reported;

- The values used to build graphs;

- The points extracted from images for analysis.

Authors do not need to submit their entire data set if only a portion of the data was used in the reported study.

If your submission does not contain these data, please either upload them as Supporting Information files or deposit them to a stable, public repository and provide us with the relevant URLs, DOIs, or accession numbers. For a list of recommended repositories, please see https://journals.plos.org/plosone/s/recommended-repositories.

If there are ethical or legal restrictions on sharing a de-identified data set, please explain them in detail (e.g., data contain potentially sensitive information, data are owned by a third-party organization, etc.) and who has imposed them (e.g., an ethics committee). Please also provide contact information for a data access committee, ethics committee, or other institutional body to which data requests may be sent. If data are owned by a third party, please indicate how others may request data access.

4. If the reviewer comments include a recommendation to cite specific previously published works, please review and evaluate these publications to determine whether they are relevant and should be cited. There is no requirement to cite these works unless the editor has indicated otherwise.

[Note: HTML markup is below. Please do not edit.]

Reviewers' comments:

Reviewer's Responses to Questions

**Comments to the Author**

1. Is the manuscript technically sound, and do the data support the conclusions?

The manuscript must describe a technically sound piece of scientific research with data that supports the conclusions. Experiments must have been conducted rigorously, with appropriate controls, replication, and sample sizes. The conclusions must be drawn appropriately based on the data presented.

Reviewer #1: Yes

Reviewer #2: Partly

**********

2. Has the statistical analysis been performed appropriately and rigorously?

Reviewer #1: Yes

Reviewer #2: I Don't Know

**********

3. Have the authors made all data underlying the findings in their manuscript fully available?

The PLOS Data policy requires authors to make all data underlying the findings described in their manuscript fully available without restriction, with rare exception (please refer to the Data Availability Statement in the manuscript PDF file). The data should be provided as part of the manuscript or its supporting information, or deposited to a public repository. For example, in addition to summary statistics, the data points behind means, medians and variance measures should be available. If there are restrictions on publicly sharing data—e.g. participant privacy or use of data from a third party—those must be specified.

Reviewer #1: Yes

Reviewer #2: Yes

**********

4. Is the manuscript presented in an intelligible fashion and written in standard English?

PLOS ONE does not copyedit accepted manuscripts, so the language in submitted articles must be clear, correct, and unambiguous. Any typographical or grammatical errors should be corrected at revision, so please note any specific errors here.

Reviewer #1: Yes

Reviewer #2: Yes

**********

5. Review Comments to the Author

Please use the space provided to explain your answers to the questions above. You may also include additional comments for the author, including concerns about dual publication, research ethics, or publication ethics. (Please upload your review as an attachment if it exceeds 20,000 characters)

Reviewer #1: Refine the Abstract and Introduction:

The abstract mentions the possibility of 5-HT depletion disturbing receptor balance. Given that this is a key part of the discussion, consider softening this or framing it more as a hypothesis generated by the data, as the study did not directly measure receptor function.

In the introduction, the transition from general migraine susceptibility to the specific concept of "intrinsic neuronal susceptibility" could be slightly smoother. A sentence explicitly stating that this study aims to define this susceptibility at the cellular level in first-order neurons would strengthen the narrative.

Clarify the Discussion:

The discussion is comprehensive but somewhat lengthy. It could be more focused. The section on other disorders (IBS, fibromyalgia) is interesting but somewhat tangential. Consider condensing this to maintain a sharp focus on migraine mechanisms.

The argument about 5-HT receptor hierarchy (page 28-29) is insightful but speculative. It would be beneficial to explicitly state that this is a proposed model based on the literature and the current findings, and that it requires direct experimental validation.

Address Limitations More Proactively:

The limitation of using only male rats is well-noted. However, when discussing the Ah-type neurons (which are female-prevalent) and their potential role, the language should be more cautious. The current text (Page 27, lines 381-383) presents a hypothesis that the study's design cannot address. Frame this more clearly as a compelling question for future research directly in female models.

Minor Technical Points:

Figure 3: Ensure the figure legend clearly states which statistical test was used for each panel (Kruskal-Wallis vs. ANOVA) and that the pairwise comparisons shown are post-hoc tests following a significant omnibus test.

Results Flow: The results section jumps between correlation analysis and group comparisons. A brief introductory sentence for each sub-section (e.g., "We first sought to understand the relationship between...") could improve readability.

Reviewer #2: The study by Saknan et al addressed the influence of serotonin depletion on the excitability of TG neurons. Althougt this is a relevant issue, several flaws reder the manuscript not aceptable for publication in its current form.The major points raised during my revision are:

1- The introduction is too long.

2- Although some parts of the methods section are very detailed, some information is missing.For example, how long after 5HT depletion the TGs were collected for culture? How long after the culture, the authors performed patch clamp?

3- There are no controls for serotonin depletion. How do the authors know that the protocol is working properly? How do they know that the depletion occurs at the TG level? How long the depletion lasts?

4- The conclusion is based solely in patch clamp experiments.

5- The discussion is too speculative. The authors present several information about the influcence of serotonin depletion in general, but they did not investigate the influence of this depletion on the TG, besides the eletrophysiological properties of the neurons. In my opinion the data is too preliminary and the study requires further experiments to show a consistent idea.

6- The authors claim that this mechanism is relevant to migraine, but they performed all the recordings in the TG of naive rats. It would be very interesting to see the influence of 5HT depletion in TG from rats subjected to a migraine model or in TG cutured neurons treated with a migarine stimulus, such as CGRP.

7- In my opinion to investigate sex differences is crucial in migraine studies, since we can have very different results depending on the sex.

**********

6. PLOS authors have the option to publish the peer review history of their article (what does this mean?). If published, this will include your full peer review and any attached files.

If you choose “no”, your identity will remain anonymous but your review may still be made public.

**Do you want your identity to be public for this peer review?**  For information about this choice, including consent withdrawal, please see our Privacy Policy.

Reviewer #1: **Yes:** Abdul Hanif Khan Yusof Khan

Reviewer #2: No

**********

[NOTE: If reviewer comments were submitted as an attachment file, they will be attached to this email and accessible via the submission site. Please log into your account, locate the manuscript record, and check for the action link "View Attachments". If this link does not appear, there are no attachment files.]

To ensure your figures meet our technical requirements, please review our figure guidelines: https://journals.plos.org/plosone/s/figures

You may also use PLOS’s free figure tool, NAAS, to help you prepare publication quality figures: https://journals.plos.org/plosone/s/figures#loc-tools-for-figure-preparation.

NAAS will assess whether your figures meet our technical requirements by comparing each figure against our figure specifications.

# Author comment: Author response to Decision Letter 1

**DOI:** 10.1371/journal.pone.0354679.r002

13 May 2026

Dear Editor,

Please find enclosed our revised manuscript entitled “Serotonin depletion amplifies migraine susceptibility through trigeminal ganglion neurons sensitization: Insights from patch clamp recordings in rat model” (PONE-D-25-46193) and a detailed response to the reviewers’ comments.

We sincerely thank you and the reviewers for the careful evaluation of our manuscript and for the constructive comments, which have significantly strengthened the quality, rigor, and clarity of our work. We have revised the manuscript extensively in response to all comments raised and provide a detailed point-by-point response to both reviewers. In particular, we have addressed the Editor’s specific concerns as follows:

1) Control of serotonin depletion in the experiments

We have clarified and strengthened the Methods, Discussion, and Limitations regarding validation of serotonin depletion. Specifically, we now provide a more detailed justification of the established PCPA depletion protocol used in this study, including literature-supported evidence demonstrating robust central and systemic serotonin depletion within the experimental time window. We also clarify the timing of tissue collection and electrophysiological recordings following PCPA treatment. While serotonin levels were not directly measured in trigeminal ganglia in the present study, this limitation is now explicitly acknowledged in the revised manuscript, and the interpretation has been appropriately tempered.

2) Relevance to migraine and the use of naïve TG neurons / consideration of migraine models or CGRP stimulation

In response to this important point and Reviewer #2’s suggestion, we have revised the manuscript to more clearly define the scope of the study as examining how serotonin depletion alters intrinsic electrophysiological susceptibility of first-order trigeminal neurons under baseline conditions, rather than directly modeling migraine pathology. We have moderated claims regarding migraine relevance accordingly. In addition, the Discussion has been expanded to explicitly discuss migraine-relevant experimental paradigms, including CGRP stimulation and established migraine models, as important future directions to test how serotonin depletion interacts with pathological triggers.

3) Response to all reviewer comments

We have carefully addressed all comments raised by both reviewers in the accompanying point-by-point response document. Revisions include substantial refinement of the Abstract, Introduction, Results flow, Discussion, limitations, statistical reporting, and interpretation of findings, with particular care taken to reduce speculative statements and align conclusions more closely with the experimental evidence.

We appreciate the opportunity to revise and improve our manuscript and hope that the revised version is now suitable for publication in PLOS ONE.

Sincerely,

Saknan Bongsebandhu-phubhakdi, Ph.D.

Corresponding Author

on behalf of all authors

Response to reviewers

Reviewer #1: Refine the Abstract and Introduction:

The abstract mentions the possibility of 5-HT depletion disturbing receptor balance. Given that this is a key part of the discussion, consider softening this or framing it more as a hypothesis generated by the data, as the study did not directly measure receptor function.

Authors’ Response:

We thank the reviewer for this important and insightful comment. We agree that the statement regarding disruption of 5-HT receptor balance should be interpreted with appropriate caution, as receptor-specific mechanisms were not directly assessed in this study.

Accordingly, we have revised the Abstract to more clearly frame this point as a hypothesis generated from our electrophysiological findings, rather than as a definitive mechanistic conclusion. This revision ensures that our interpretation remains consistent with the scope of the data while still highlighting a biologically plausible implication that may guide future investigations.

Changes in manuscript:

The relevant sentence in the Abstract has been revised to explicitly reflect a hypothesis-generating interpretation rather than a causal inference (page 2, lines 22–27).

In the introduction, the transition from general migraine susceptibility to the specific concept of "intrinsic neuronal susceptibility" could be slightly smoother. A sentence explicitly stating that this study aims to define this susceptibility at the cellular level in first-order neurons would strengthen the narrative.

Authors’ Response:

We appreciate the reviewer’s constructive suggestion. We agree that a clearer conceptual transition would improve the readability and logical flow of the Introduction.

In response, we have revised the relevant section to provide a more gradual progression from general migraine susceptibility to the specific concept of “intrinsic neuronal susceptibility.” Importantly, we have added a clarifying sentence explicitly stating that this study aims to define this susceptibility at the cellular level of first-order trigeminal ganglion neurons. This addition strengthens the conceptual framework and more clearly positions our study within the broader context of migraine pathophysiology.

Changes in manuscript:

A new sentence has been added to the Introduction to explicitly define the study objective and improve conceptual continuity (page 4–5, lines 45–77; page 7, lines 134–143).

Clarify the Discussion:

The discussion is comprehensive but somewhat lengthy. It could be more focused. The section on other disorders (IBS, fibromyalgia) is interesting but somewhat tangential. Consider condensing this to maintain a sharp focus on migraine mechanisms.

Authors’ Response:

We thank the reviewer for this constructive and helpful suggestion. We agree that improving the focus and conciseness of the Discussion strengthens the overall clarity of the manuscript.

Accordingly, we have revised the Discussion to achieve a more streamlined and migraine-centered narrative. In particular, the section addressing other serotonin-related disorders, including irritable bowel syndrome (IBS) and fibromyalgia, has been substantially condensed. While these conditions remain briefly mentioned to contextualize the broader relevance of serotonergic dysregulation, excessive detail has been removed to maintain a sharper focus on migraine-specific mechanisms.

These revisions improve readability while preserving the conceptual contribution of our findings within the context of migraine pathophysiology.

Changes in manuscript:

The Discussion section has been condensed, with the section on IBS and fibromyalgia substantially shortened and refocused (page 27, lines 536–543).

The argument about 5-HT receptor hierarchy (page 28-29) is insightful but speculative. It would be beneficial to explicitly state that this is a proposed model based on the literature and the current findings, and that it requires direct experimental validation.

Authors’ Response:

We appreciate the reviewer’s insightful comment. We agree that the proposed 5-HT receptor hierarchy represents a conceptual interpretation rather than a directly tested mechanism in the present study.

In response, we have revised the relevant section to explicitly frame this as a hypothesis-driven model derived from existing literature in combination with our electrophysiological findings. We have also clearly stated that this model requires direct experimental validation, particularly through receptor-specific and molecular investigations.

This clarification ensures appropriate distinction between data-driven conclusions and broader mechanistic interpretation, thereby improving the scientific rigor and transparency of the Discussion.

Changes in manuscript:

The section discussing 5-HT receptor hierarchy has been revised to explicitly identify it as a proposed model and to highlight the need for direct experimental validation (page 25, lines 487–505).

Address Limitations More Proactively:

The limitation of using only male rats is well-noted. However, when discussing the Ah-type neurons (which are female-prevalent) and their potential role, the language should be more cautious. The current text (Page 27, lines 381-383) presents a hypothesis that the study's design cannot address. Frame this more clearly as a compelling question for future research directly in female models.

Authors’ Response:

We thank the reviewer for this important and insightful comment. We fully agree that the discussion of Ah-type neurons requires more cautious interpretation, particularly given the male-only design of the present study.

Accordingly, we have revised the text to clearly acknowledge that, although our findings suggest that large-sized TG neurons may shift toward a more nociceptive-like electrophysiological profile following 5-HT depletion, the specific involvement of Ah-type neurons—predominantly characterized in female models—cannot be determined from our data.

This point has now been explicitly reframed as a key question for future investigation. We emphasize the importance of studies in female models to directly assess the role of Ah-type neurons and to better understand sex-specific mechanisms in migraine pathophysiology.

These revisions ensure that our interpretation remains aligned with the experimental scope while highlighting a meaningful direction for future research.

Changes in manuscript:

The relevant section has been revised to adopt a more cautious interpretation and to reposition the role of Ah-type neurons as a question for future studies in female models (page 23, lines 444–451).

Minor Technical Points:

Figure 3: Ensure the figure legend clearly states which statistical test was used for each panel (Kruskal-Wallis vs. ANOVA) and that the pairwise comparisons shown are post-hoc tests following a significant omnibus test.

Authors’ response:

We thank the reviewer for this helpful and important suggestion. We agree that clear reporting of statistical methods in figure legends is essential for transparency and reproducibility.

Accordingly, we have revised the Figure 3 legend to explicitly specify the statistical test applied to each parameter, indicating whether a Kruskal–Wallis test or one-way ANOVA was used based on data distribution. We have also clarified that the pairwise comparisons shown in the figure represent post-hoc analyses performed only following a significant omnibus test.

These revisions improve the clarity and interpretability of the statistical presentation.

Changes in manuscript:

The Figure 3 legend has been revised to explicitly describe the statistical tests used for each panel and the conditions under which post-hoc comparisons were conducted (page 19, lines 364–367).

Results Flow: The results section jumps between correlation analysis and group comparisons. A brief introductory sentence for each sub-section (e.g., "We first sought to understand the relationship between...") could improve readability.

Authors’ response:

We appreciate the reviewer’s insightful suggestion. We agree that improving the structural organization of the Results section enhances readability and comprehension.

In response, we have reorganized the Results into clearly defined subsections that separate correlation analyses from intergroup comparisons. Additionally, brief introductory sentences have been incorporated at the beginning of each subsection to clarify the analytical objective and guide the reader through the progression of the results.

These revisions improve the logical flow and overall clarity of the Results section.

Changes in manuscript:

The Results section has been restructured into subsections with added introductory statements to improve clarity and narrative flow (page 12, lines 252–254; page 13, lines 276–278; page 15, lines 303–306; page 16, lines 333–335).

Reviewer #2: The study by Saknan et al addressed the influence of serotonin depletion on the excitability of TG neurons. Althougt this is a relevant issue, several flaws reder the manuscript not aceptable for publication in its current form.The major points raised during my revision are:

1- The introduction is too long.

Authors’ response:

We thank the reviewer for this constructive comment. We agree that the original Introduction was overly detailed, which may have affected clarity and focus.

In response, we have substantially revised and streamlined the Introduction to improve conciseness and readability. Redundant background information has been removed, and the section has been reorganized to more clearly emphasize the key knowledge gap and the rationale for the present study. In particular, we have tightened the narrative to focus more directly on the role of serotonin depletion in trigeminal ganglion neuronal excitability and its relevance to migraine pathophysiology.

As a result of these revisions, the length of the Introduction has been reduced from 862 to 654 words (approximately a 24% reduction), leading to a more focused and accessible presentation.

These revisions result in a more focused and accessible Introduction that better guides the reader to the objectives of the study.

Changes in manuscript:

The Introduction has been substantially condensed and reorganized to improve clarity, focus, and narrative flow (page 3, lines 45–77; page 7, lines 134–143).

2- Although some parts of the methods section are very detailed, some information is missing.For example, how long after 5HT depletion the TGs were collected for culture? How long after the culture, the authors performed patch clamp?

Authors’ response:

We thank the reviewer for this important and insightful comment. We agree that precise timing of experimental procedures is essential for clarity and reproducibility.

In response, we have revised the Methods section to explicitly clarify the temporal sequence of the experimental protocol. Specifically, 5-HT depletion was induced using a well-established PCPA regimen (100 mg/kg, i.p., for three consecutive days), which has been shown to produce substantial central and systemic serotonin depletion within 24 hours after the final injection [1].

In the present study, trigeminal ganglia were harvested 24 hours after the final PCPA administration, corresponding to a time point at which serotonin depletion is well established based on prior literature. Following enzymatic dissociation, TG neurons were cultured for 18–24 hours prior to electrophysiological recordings, and whole-cell patch-clamp experiments were performed within this defined time window.

These details have now been explicitly incorporated into the revised Methods section to improve transparency and reproducibility.

Changes in manuscript:

The Methods section has been revised to clearly specify the timing of TG harvesting and electrophysiological recordings following 5-HT depletion (page 9, lines 183–184). Additionally, we have added new reference #36 (page 36, lines 725–726).

3- There are no controls for serotonin depletion. How do the authors know that the protocol is working properly? How do they know that the depletion occurs at the TG level? How long the depletion lasts?

Authors’ response:

We thank the reviewer for this critical and important comment. We agree that validation of serotonin depletion is essential for the interpretation of our findings.

In the present study, 5-HT depletion was induced using a well-established PCPA protocol (100 mg/kg, i.p., for three consecutive days), which has been extensively validated across multiple studies to produce robust and reproducible depletion of serotonin in both central and peripheral compartments [1–3]. Specifically, this regimen has been shown to reduce brain 5-HT levels to approximately 10–20% of control within 24 hours after the final injection.

Importantly, PCPA inhibits tryptophan hydroxylase systemically, leading to global suppression of serotonin synthesis. Previous studies have demonstrated that this results not only in central depletion but also in marked reductions in circulating (whole blood and plasma) serotonin, as well as in peripheral tissues such as the spleen and gastrointestinal tract [1,4]. Given that trigeminal ganglion neurons are part of the peripheral nervous system and are directly exposed to systemic biochemical change

Attachment Submitted filename: PONE-D-25-46193_Response to Reviewers.docx

# Peer review: Decision Letter 1

**DOI:** 10.1371/journal.pone.0354679.r003

**Authors:** Paulo Ho

28 May 2026

PONE-D-25-46193R1

Serotonin depletion amplifies migraine susceptibility through trigeminal ganglion neurons sensitization: Insights from patch clamp recordings in rat model

PLOS One

Dear Dr. Bongsebandhu-phubhakdi,

Thank you for submitting your manuscript to PLOS ONE. After careful consideration, we feel that it has merit but does not fully meet PLOS ONE’s publication criteria as it currently stands. Therefore, we invite you to submit a revised version of the manuscript that addresses the points raised during the review process.

Please submit your revised manuscript by Jul 12 2026 11:59PM. If you will need more time than this to complete your revisions, please reply to this message or contact the journal office at plosone@plos.org. When you're ready to submit your revision, log on to https://www.editorialmanager.com/pone/ and select the 'Submissions Needing Revision' folder to locate your manuscript file.

Please include the following items when submitting your revised manuscript:

- A letter that responds to each point raised by the academic editor and reviewer(s). You should upload this letter as a separate file labeled 'Response to Reviewers'.
- A marked-up copy of your manuscript that highlights changes made to the original version. You should upload this as a separate file labeled 'Revised Manuscript with Track Changes'.
- An unmarked version of your revised paper without tracked changes. You should upload this as a separate file labeled 'Manuscript'.

If you would like to make changes to your financial disclosure, please include your updated statement in your cover letter. Guidelines for resubmitting your figure files are available below the reviewer comments at the end of this letter.

If applicable, we recommend that you deposit your laboratory protocols in protocols.io to enhance the reproducibility of your results. Protocols.io assigns your protocol its own identifier (DOI) so that it can be cited independently in the future. For instructions see: https://journals.plos.org/plosone/s/submission-guidelines#loc-laboratory-protocols. Additionally, PLOS ONE offers an option for publishing peer-reviewed Lab Protocol articles, which describe protocols hosted on protocols.io. Read more information on sharing protocols at https://plos.org/protocols?utm_medium=editorial-email&utm_source=authorletters&utm_campaign=protocols.

As the corresponding author, your ORCID iD is verified in the submission system and will appear in the published article. PLOS supports the use of ORCID, and we encourage all coauthors to register for an ORCID iD and use it as well. Please encourage your coauthors to verify their ORCID iD within the submission system before final acceptance, as unverified ORCID iDs will not appear in the published article. *Only*  the individual author can complete the verification step; PLOS staff *cannot*  verify ORCID iDs on behalf of authors.

We look forward to receiving your revised manuscript.

Kind regards,

Paulo Lee Ho, Ph.D.

Academic Editor

PLOS One

Journal Requirements:

If the reviewer comments include a recommendation to cite specific previously published works, please review and evaluate these publications to determine whether they are relevant and should be cited. There is no requirement to cite these works unless the editor has indicated otherwise.

Please review your reference list to ensure that it is complete and correct. If you have cited papers that have been retracted, please include the rationale for doing so in the manuscript text, or remove these references and replace them with relevant current references. Any changes to the reference list should be mentioned in the rebuttal letter that accompanies your revised manuscript. If you need to cite a retracted article, indicate the article’s retracted status in the References list and also include a citation and full reference for the retraction notice.

[Note: HTML markup is below. Please do not edit.]

Reviewers' comments:

Reviewer's Responses to Questions

**Comments to the Author**

1. If the authors have adequately addressed your comments raised in a previous round of review and you feel that this manuscript is now acceptable for publication, you may indicate that here to bypass the “Comments to the Author” section, enter your conflict of interest statement in the “Confidential to Editor” section, and submit your "Accept" recommendation.

Reviewer #1: All comments have been addressed

Reviewer #2: All comments have been addressed

**********

2. Is the manuscript technically sound, and do the data support the conclusions?

The manuscript must describe a technically sound piece of scientific research with data that supports the conclusions. Experiments must have been conducted rigorously, with appropriate controls, replication, and sample sizes. The conclusions must be drawn appropriately based on the data presented.

Reviewer #1: Yes

Reviewer #2: Yes

**********

3. Has the statistical analysis been performed appropriately and rigorously?

Reviewer #1: Yes

Reviewer #2: I Don't Know

**********

4. Have the authors made all data underlying the findings in their manuscript fully available?

The PLOS Data policy requires authors to make all data underlying the findings described in their manuscript fully available without restriction, with rare exception (please refer to the Data Availability Statement in the manuscript PDF file). The data should be provided as part of the manuscript or its supporting information, or deposited to a public repository. For example, in addition to summary statistics, the data points behind means, medians and variance measures should be available. If there are restrictions on publicly sharing data—e.g. participant privacy or use of data from a third party—those must be specified.

Reviewer #1: Yes

Reviewer #2: Yes

**********

5. Is the manuscript presented in an intelligible fashion and written in standard English?

PLOS ONE does not copyedit accepted manuscripts, so the language in submitted articles must be clear, correct, and unambiguous. Any typographical or grammatical errors should be corrected at revision, so please note any specific errors here.

Reviewer #1: Yes

Reviewer #2: Yes

**********

6. Review Comments to the Author

Please use the space provided to explain your answers to the questions above. You may also include additional comments for the author, including concerns about dual publication, research ethics, or publication ethics. (Please upload your review as an attachment if it exceeds 20,000 characters)

Reviewer #1: Strengths:

You have thoroughly addressed previous reviewer concerns, particularly regarding validation of serotonin depletion, timing of experiments, and the speculative nature of receptor interpretations.

The revision now clearly frames the 5-HT receptor discussion as hypothesis-generating, which is appropriate.

The limitations section is more comprehensive, acknowledging lack of direct 5-HT measurement in TG, absence of female models, and the reductionist approach.

Remaining issues to consider before final acceptance:

Redundancy and length – The Introduction and Discussion still contain some repetitive passages (e.g., the concept of "intrinsic neuronal susceptibility" is defined multiple times). Consider a final pruning for conciseness.

Minor error in Table 1 – The footnote "p-value Pearson's Chi- square test" is incomplete; specify it is for bursting profiles.

References – The citation numbers in the text appear to have shifted; ensure consistency with the reference list.

Figure legends – Figure 3 legend correctly specifies Kruskal-Wallis vs ANOVA, but ensure the figure panels (A–I) are clearly labeled in the submitted figure file.

Language – A few awkward phrasing remain (e.g., "the experiment raises the possibility that 5-HT depletion may also indirectly disturb" in abstract is repetitive). A light copyedit would help.

Overall, the scientific content is sound, the methodology is appropriate, and the conclusions are now appropriately tempered. The study provides a useful electrophysiological foundation for understanding serotonergic modulation of trigeminal ganglion neurons.

Reviewer #2: (No Response)

**********

7. PLOS authors have the option to publish the peer review history of their article (what does this mean?). If published, this will include your full peer review and any attached files.

If you choose “no”, your identity will remain anonymous but your review may still be made public.

**Do you want your identity to be public for this peer review?**  For information about this choice, including consent withdrawal, please see our Privacy Policy.

Reviewer #1: No

Reviewer #2: No

**********

[NOTE: If reviewer comments were submitted as an attachment file, they will be attached to this email and accessible via the submission site. Please log into your account, locate the manuscript record, and check for the action link "View Attachments". If this link does not appear, there are no attachment files.]

To ensure your figures meet our technical requirements, please review our figure guidelines: https://journals.plos.org/plosone/s/figures

You may also use PLOS’s free figure tool, NAAS, to help you prepare publication quality figures: https://journals.plos.org/plosone/s/figures#loc-tools-for-figure-preparation.

NAAS will assess whether your figures meet our technical requirements by comparing each figure against our figure specifications.

# Author comment: Author response to Decision Letter 2

**DOI:** 10.1371/journal.pone.0354679.r004

29 Jun 2026

Response to reviewers

Reviewer #1:

Redundancy and length – The Introduction and Discussion still contain some repetitive passages (e.g., the concept of "intrinsic neuronal susceptibility" is defined multiple times). Consider a final pruning for conciseness.

Authors’ Response:

We thank the reviewer for this helpful suggestion. We carefully reviewed the manuscript and further improved its conciseness by removing repetitive wording, particularly where the concept of intrinsic neuronal susceptibility was repeatedly described. In several instances, we replaced repetitive expressions with more precise terminology (e.g., neuronal hyperexcitability, intrinsic electrophysiological alterations, and altered baseline excitability) while preserving the intended scientific meaning.

Changes in manuscript:

The relevant wordings in the text been revised (Introduction: page 5, lines 73–74; page 6, lines 83–86, Discussion: page 17, lines 311–312; page 18, lines 332–333; page 19, lines 359–360; page 21, lines 415–416; page 21, lines 420; page 23, lines 446–447; page 23, lines 460–462).

Minor error in Table 1 – The footnote "p-value Pearson's Chi- square test" is incomplete; specify it is for bursting profiles.

Authors’ Response:

We thank the reviewer for this helpful comment. We have revised the Table 1 footnote to clarify that the reported Pearson's Chi-square test was used to compare the distribution of bursting profiles (single-spike vs. multiple-spike neurons) between groups, thereby improving the clarity and completeness of the statistical description.

Changes in manuscript:

The relevant sentence in the footnote Table 1 has been revised (page 11, lines 213–215).

References – The citation numbers in the text appear to have shifted; ensure consistency with the reference list.

Authors’ Response:

We thank the reviewer for this careful observation. We have thoroughly checked all in-text citations and verified their consistency with the revised reference list. Any numbering inconsistencies introduced during the previous revision have now been corrected.

Figure legends – Figure 3 legend correctly specifies Kruskal-Wallis vs ANOVA, but ensure the figure panels (A–I) are clearly labeled in the submitted figure file.

Authors’ Response:

We thank the reviewer for this helpful comment. We have carefully checked the submitted Figure 3 file and ensured that all panels (A–I) are clearly labeled and correspond consistently with the descriptions provided in the figure legend.

Changes in manuscript:

The relevant sentence in the figure legends of Figure 3 has been revised (page 16, lines 305–308).

Language – A few awkward phrasing remain (e.g., "the experiment raises the possibility that 5-HT depletion may also indirectly disturb" in abstract is repetitive). A light copyedit would help.

Authors’ Response:

We thank the reviewer for this helpful comment. The manuscript has undergone an additional round of careful language editing to improve clarity, conciseness, and readability. We have also corrected several awkward or repetitive phrases throughout the manuscript, including in the Abstract, Introduction, and Discussion.

Overall, the scientific content is sound, the methodology is appropriate, and the conclusions are now appropriately tempered. The study provides a useful electrophysiological foundation for understanding serotonergic modulation of trigeminal ganglion neurons.

Authors’ Response:

We sincerely thank the reviewer for the careful evaluation of our manuscript throughout the review process and for this encouraging final assessment. We greatly appreciate the reviewer’s constructive comments, which have helped us substantially improve the clarity, rigor, and balance of the manuscript. We are pleased that the revised version is now considered scientifically sound and that our study provides a useful electrophysiological foundation for understanding serotonergic modulation of trigeminal ganglion neurons.

Attachment Submitted filename: PONE-D-25-46193_Response_to_Reviewers_auresp_2.docx

# Peer review: Decision Letter 2

**DOI:** 10.1371/journal.pone.0354679.r005

**Authors:** Paulo Ho

13 Jul 2026

Serotonin depletion amplifies migraine susceptibility through trigeminal ganglion neurons sensitization: Insights from patch clamp recordings in rat model

PONE-D-25-46193R2

Dear Dr. Bongsebandhu-phubhakdi,

We’re pleased to inform you that your manuscript has been judged scientifically suitable for publication and will be formally accepted for publication once it meets all outstanding technical requirements.

Within one week, you’ll receive an e-mail detailing the required amendments. When these have been addressed, you’ll receive a formal acceptance letter and your manuscript will be scheduled for publication.

An invoice will be generated when your article is formally accepted. Please note, if your institution has a publishing partnership with PLOS and your article meets the relevant criteria, all or part of your publication costs will be covered. Please make sure your user information is up-to-date by logging into Editorial Manager at Editorial Manager® and clicking the ‘Update My Information' link at the top of the page. For questions related to billing, please contact billing support.

If your institution or institutions have a press office, please notify them about your upcoming paper to help maximize its impact. If they’ll be preparing press materials, please inform our press team as soon as possible -- no later than 48 hours after receiving the formal acceptance. Your manuscript will remain under strict press embargo until 2 pm Eastern Time on the date of publication. For more information, please contact onepress@plos.org.

Kind regards,

Paulo Lee Ho, Ph.D.

Academic Editor

PLOS One

Additional Editor Comments (optional):

Reviewers' comments:

Reviewer's Responses to Questions

**Comments to the Author**

1. If the authors have adequately addressed your comments raised in a previous round of review and you feel that this manuscript is now acceptable for publication, you may indicate that here to bypass the “Comments to the Author” section, enter your conflict of interest statement in the “Confidential to Editor” section, and submit your "Accept" recommendation.

Reviewer #2: All comments have been addressed

**********

2. Is the manuscript technically sound, and do the data support the conclusions?

The manuscript must describe a technically sound piece of scientific research with data that supports the conclusions. Experiments must have been conducted rigorously, with appropriate controls, replication, and sample sizes. The conclusions must be drawn appropriately based on the data presented.

Reviewer #2: Yes

**********

3. Has the statistical analysis been performed appropriately and rigorously?

Reviewer #2: I Don't Know

**********

4. Have the authors made all data underlying the findings in their manuscript fully available?

The PLOS Data policy requires authors to make all data underlying the findings described in their manuscript fully available without restriction, with rare exception (please refer to the Data Availability Statement in the manuscript PDF file). The data should be provided as part of the manuscript or its supporting information, or deposited to a public repository. For example, in addition to summary statistics, the data points behind means, medians and variance measures should be available. If there are restrictions on publicly sharing data—e.g. participant privacy or use of data from a third party—those must be specified.

Reviewer #2: Yes

**********

5. Is the manuscript presented in an intelligible fashion and written in standard English?

PLOS ONE does not copyedit accepted manuscripts, so the language in submitted articles must be clear, correct, and unambiguous. Any typographical or grammatical errors should be corrected at revision, so please note any specific errors here.

Reviewer #2: Yes

**********

6. Review Comments to the Author

Please use the space provided to explain your answers to the questions above. You may also include additional comments for the author, including concerns about dual publication, research ethics, or publication ethics. (Please upload your review as an attachment if it exceeds 20,000 characters)

Reviewer #2: The authors had adressed all my comments in the first revision of the manuscript. I have no additional comments.

**********

7. PLOS authors have the option to publish the peer review history of their article (what does this mean?). If published, this will include your full peer review and any attached files.

If you choose “no”, your identity will remain anonymous but your review may still be made public.

**Do you want your identity to be public for this peer review?**  For information about this choice, including consent withdrawal, please see our Privacy Policy.

Reviewer #2: No

**********

# Editor report: Acceptance letter

**DOI:** 10.1371/journal.pone.0354679.r006

**Authors:** Paulo Ho

PONE-D-25-46193R2

PLOS One

Dear Dr. Bongsebandhu-phubhakdi,

I'm pleased to inform you that your manuscript has been deemed suitable for publication in PLOS One. Congratulations! Your manuscript is now being handed over to our production team.

At this stage, our production department will prepare your paper for publication. This includes ensuring the following:

* All references, tables, and figures are properly cited

* All relevant supporting information is included in the manuscript submission,

* There are no issues that prevent the paper from being properly typeset

You will receive further instructions from the production team, including instructions on how to review your proof when it is ready. Please keep in mind that we are working through a large volume of accepted articles, so please give us a few days to review your paper and let you know the next and final steps.

Lastly, if your institution or institutions have a press office, please let them know about your upcoming paper now to help maximize its impact. If they'll be preparing press materials, please inform our press team within the next 48 hours. Your manuscript will remain under strict press embargo until 2 pm Eastern Time on the date of publication. For more information, please contact onepress@plos.org.

You will receive an invoice from PLOS for your publication fee after your manuscript has reached the completed accept phase. If you receive an email requesting payment before acceptance or for any other service, this may be a phishing scheme. Learn how to identify phishing emails and protect your accounts at https://explore.plos.org/phishing.

If we can help with anything else, please email us at customercare@plos.org.

Thank you for submitting your work to PLOS ONE and supporting open access.

Kind regards,

PLOS ONE Editorial Office Staff

on behalf of

Dr. Paulo Lee Ho

Academic Editor

PLOS One
